# Propolis effects on blood sugar and lipid metabolism, inflammatory indicators, and oxidative stress in people with type 2 diabetes: a systematic review and meta-analysis

**DOI:** 10.3389/fnut.2025.1653730

**Published:** 2025-10-09

**Authors:** Yihua Zhang, Shuo Ding, Wenjing Li, Xiumei Wang, Jie Lv, Qingmei Niu, Qian Zhang

**Affiliations:** ^1^School of Nursing, Shanxi University of Chinese Medicine, Yuci, China; ^2^Department of Central Operating Room, Shanxi Bethune Hospital Shanxi Academy of Medical Sciences, Tongji Shanxi Hospital, Third Hospital of Shanxi Medical University, Taiyuan, China; ^3^Department of Nursing, Shanxi Bethune Hospital, Shanxi Academy of Medical Sciences, Tongji Shanxi Hospital, Third Hospital of Shanxi Medical University, Taiyuan, China

**Keywords:** propolis, type 2 diabetes mellitus, glycolipid metabolism, inflammatory markers, oxidative stress, systematic review

## Abstract

**Background:**

Type 2 Diabetes Mellitus (T2DM) poses a significant global health challenge. Propolis, a natural bioactive compound, is proposed to modulate glucose and lipid metabolism and exert anti-inflammatory effects. However, previous reviews have limited scope, and the effects of propolis on T2DM remain debated, particularly concerning lipid profiles, glycemic control, inflammation, and oxidative stress.

**Methods:**

A systematic search was conducted across Chinese National Knowledge Infrastructure (CNKI), VIP, SinoMed, Wanfang Data, PubMed, Cochrane Library, Embase, Scopus, and Web of Science, with the search time limit set from the establishment of the databases to 20 May 2025. Study quality was assessed using the Cochrane Risk of Bias Assessment Tool version 2 (ROB 2); evidence quality was evaluated via the Grading of Recommendations, Assessment, Development, and Evaluation (GRADE) approach; and meta-analysis was performed using RevMan 5.4.

**Results:**

In total, 12 randomized controlled trials (RCTs) with 731 participants were included in this study. Propolis supplementation significantly increased high-density lipoprotein cholesterol (HDL-C) levels (mean difference (MD) = 0.13, 95% CI 0.10–0.16, *p* < 0.00001), and reduced low-density lipoprotein cholesterol (LDL-C) (MD = −0.32, 95% CI: −0.56 to −0.08; *p* = 0.009) and triglyceride (TG) levels (MD = −0.15, 95% CI: −0.30 to −0.01; *p* = 0.04). It also improved glycemic control, lowering fasting blood sugar (FBS) (MD = −1.13, 95% CI: −2.00 to −0.27, *p* = 0.01), homeostasis model assessment of insulin resistance (HOMA-IR) (MD = −0.95, 95% CI: −1.36 to −0.55, *p* < 0.00001), and glycosylated hemoglobin (HbA1c) (MD = −0.44, 95% CI: −0.78 to −0.11, *p* = 0.01). Furthermore, propolis significantly reduced C-reactive protein (CRP) (MD = −2.68, 95% CI: −3.48 to −1.89, *p* < 0.00001). However, no significant effects were observed for total cholesterol (TC), tumor necrosis factor-alpha (TNF-*α*), interleukin-6 (IL-6), superoxide dismutase (SOD), or malondialdehyde (MDA).

**Conclusion:**

Propolis may improve lipid and glucose profiles and reduce inflammation in T2DM. While current evidence does not confirm significant effects on oxidative stress markers, considering the limitations of existing clinical studies and positive basic research findings, its potential antioxidant effects require validation through high-quality RCTs.

**Systematic review resistration:**

This study was registered with PROSPERO (registration number: CRD42024577722) https://www.crd.york.ac.uk/PROSPERO/#loginpage.

## Introduction

Type 2 Diabetes Mellitus (T2DM) is a chronic metabolic disorder characterized by insulin resistance and dysfunction of pancreatic *β*-cells. It accounts for over 90% of global diabetes cases and exhibits a trend toward younger onset, and represents a major public health burden (1, 2). Globally, 537 million adults live with diabetes, a number projected to exceed 700 million by 2045. Approximately 40% of these individuals may develop chronic kidney disease (CKD) (3). The World Health Organization (WHO) reports that over half of patients do not adhere to regular medication, particularly in low- and middle-income countries with poor treatment coverage, increasing risks for complications like blindness, renal failure, and cardiovascular disease (4).

Current T2DM management relies heavily on pharmacological glucose control, but this approach carries significant safety concerns (5–8). For example, a regional study in Asia found that 35.8% of T2DM patients using oral hypoglycemic agents experienced hypoglycemia within 6 months (6). Sulfonylurea medications can impair hypoglycemia awareness and potentially cause severe complications such as cognitive dysfunction and arrhythmias (7). Thiazolidinediones are also linked to an increased risk of fractures and bladder cancer (9). Therefore, exploring safe, cost-effective, and efficient complementary therapies for T2DM is crucial.

Various plant-derived bioactive compounds have been investigated for T2DM adjunctive treatment, yet many show limited efficacy or practical application issues. For instance, curcumin has notable anti-inflammatory and antioxidant properties and demonstrated hypoglycemic potential in clinical trials, but its low oral bioavailability severely restricts clinical translation (10). Similarly, while okra may temporarily lower fasting blood glucose, it does not significantly improve glycated hemoglobin (HbA1c) levels (11). In contrast, propolis, as a natural nutraceutical with historical medicinal applications (12), appears more promising for T2DM intervention. It shows potential for improving insulin resistance, protecting pancreatic *β*-cell function, and has comparatively better absorption/utilization (13), possibly addressing current treatment limitations.

Propolis (14) is a natural substance collected by bees from plant sources like bark crevices and leaf buds, used by humans since ancient times and documented in pharmacopeias 4 centuries ago (15, 16). It is rich in beneficial bioactive non-nutrients, including flavonoids, polyphenols, and terpenes, with flavonoids being the most abundant and primary bioactive components (17, 18). It finds numerous applications in the treatment of various diseases (13, 19–22). Propolis has numerous applications, including antibacterial, antiviral, and anti-inflammatory effects, improving gut microbiota, promoting wound healing, and immune modulation (22–24).

While previous systematic reviews have investigated propolis effects on specific parameters such as blood glucose or lipids (25–28), they were often limited by a narrow focus on single outcomes or considerable heterogeneity among included populations. Consequently, a comprehensive assessment of its efficacy specifically in patients with T2DM is still lacking. Moreover, existing studies examining the effects of propolis on blood lipids (29–34), blood glucose (31–35), inflammatory markers (31, 32), and oxidative stress markers (35–38) in T2DM have reported inconsistent results. To address these gaps, this study systematically reviewed randomized controlled trials (RCTs) from Chinese and English databases. It aims to comprehensively evaluate the overall effects of propolis supplementation on multiple metabolic indicators in T2DM and to analyze the influence of factors such as dosage and intervention duration on therapeutic outcomes, thereby providing robust evidence to support its clinical application in diabetes management.

## Materials and methods

This study was registered with PROSPERO (registration number: CRD42024577722).^1^ During the compilation of this manuscript, it strictly abided by the guidelines outlined in the Primary Reporting Items for Systematic Reviews and Meta-Analyses (PRISMA) (39).

### Inclusion criteria

Participants: Adults aged ≥18 years diagnosed with T2DM based on clinical criteria (40).

Interventions: The intervention group was treated with propolis (capsules, tablets, etc.).

Control: The control group received a conventional intervention or placebo.

Outcome: The primary outcomes were as follows: lipid indicators, including low-density lipoprotein cholesterol (LDL-C), total cholesterol (TC), triglycerides (TG), and high-density lipoprotein cholesterol (HDL-C); and glycemic markers, namely fasting blood sugar (FBS), glycosylated hemoglobin (HbA1c), and homeostasis model assessment of insulin resistance (HOMA-IR). The secondary outcomes were as follows: inflammatory markers such as C-reactive protein (CRP), tumor necrosis factor alpha (TNF-*α*), interleukin-6 (IL-6); and oxidative stress markers, including superoxide dismutase (SOD) and malondialdehyde (MDA). The studies must provide data on at least one outcome parameter.

Study design: Randomized controlled trials (RCTs).

### Exclusion criteria

(1) Studies on propolis combined with other drugs/active substances; (2) Studies that are replications of published studies; (3) Studies for which the full text or incomplete data were unavailable; (4) Reviews, conference abstracts, animal experimental studies, etc.

### Search strategy

Two researchers independently searched nine databases (China National Knowledge Infrastructure (CNKI), Wanfang Data, VIP, SinoMed, PubMed, Cochrane Library, Embase, Scopus, and Web of Science) from inception to 20 May 2025. A hybrid search strategy combining subject headings with free terms was employed. The detailed search strategy is provided in Additional File 1.

### Literature screening and data extraction

Two researchers independently conducted literature searches and imported the retrieved records into EndNote 21 reference management software to remove duplicates. Subsequently, titles and abstracts were screened to exclude irrelevant studies. Full-texts were then assessed against the inclusion and exclusion criteria to identify eligible studies. Data extraction was performed independently by the two researchers, with information including publication year, first author, sample size, participant characteristics, propolis dosage, propolis formulation, intervention duration, and outcome measures.

### Literature quality assessment

The quality of the included studies was assessed using the Cochrane Risk of Bias tool for Randomized Trials, version 2 (RoB 2) (39). The evaluation dimensions included the randomization process, deviation from the intended interventions, missing outcome data, measurement of outcomes, and selection of reported results. The assessment results were presented in the form of a risk of bias graph. Based on the risk of bias results, each study was categorized as “high risk,” “some concerns,” or “low risk.” In cases of disagreement during this process, a third researcher acted as an arbiter to reach a final consensus.

### Evidence quality assessment

The certainty of evidence was evaluated using the Grading of Recommendations, Assessment, Development, and Evaluations (GRADE) framework. According to GRADE, the initial quality of evidence from randomized controlled trials (RCTs) was classified as high. This rating could be downgraded to moderate, low, or very low if limitations were identified in any of the five domains: Risk of bias, Inconsistency, Indirectness, Imprecision, or Publication bias. Conversely, evidence quality could be upgraded in cases of substantial effect magnitudes or observed dose–response gradients. Disagreements during assessment were resolved through arbitration by a third researcher to achieve consensus.

### Data analysis methods

Meta-analysis of included studies was performed using Review Manager 5.4. Results were presented in forest plots. Heterogeneity was assessed; a fixed-effects model was utilized if *p* ≥ 0.1 and I^2^ ≤ 50%; otherwise, a random-effects model was adopted. To identify potential sources of heterogeneity, subgroup analyses were performed based on intervention dosage and duration. A sensitivity analysis, in which each study was sequentially removed, confirmed the robustness of the pooled estimates. Publication bias was assessed with funnel plots and Egger’s test for outcomes involving 10 or more studies; for outcomes with fewer studies, these tests were considered underpowered. All outcome measures were standardized continuous variables, and the effect size was expressed as the weighted mean difference (MD) with 95% confidence interval (CI). A *p*-value less than 0.05 was considered statistically significant.

## Results

### Literature search results

**Figure 1:**
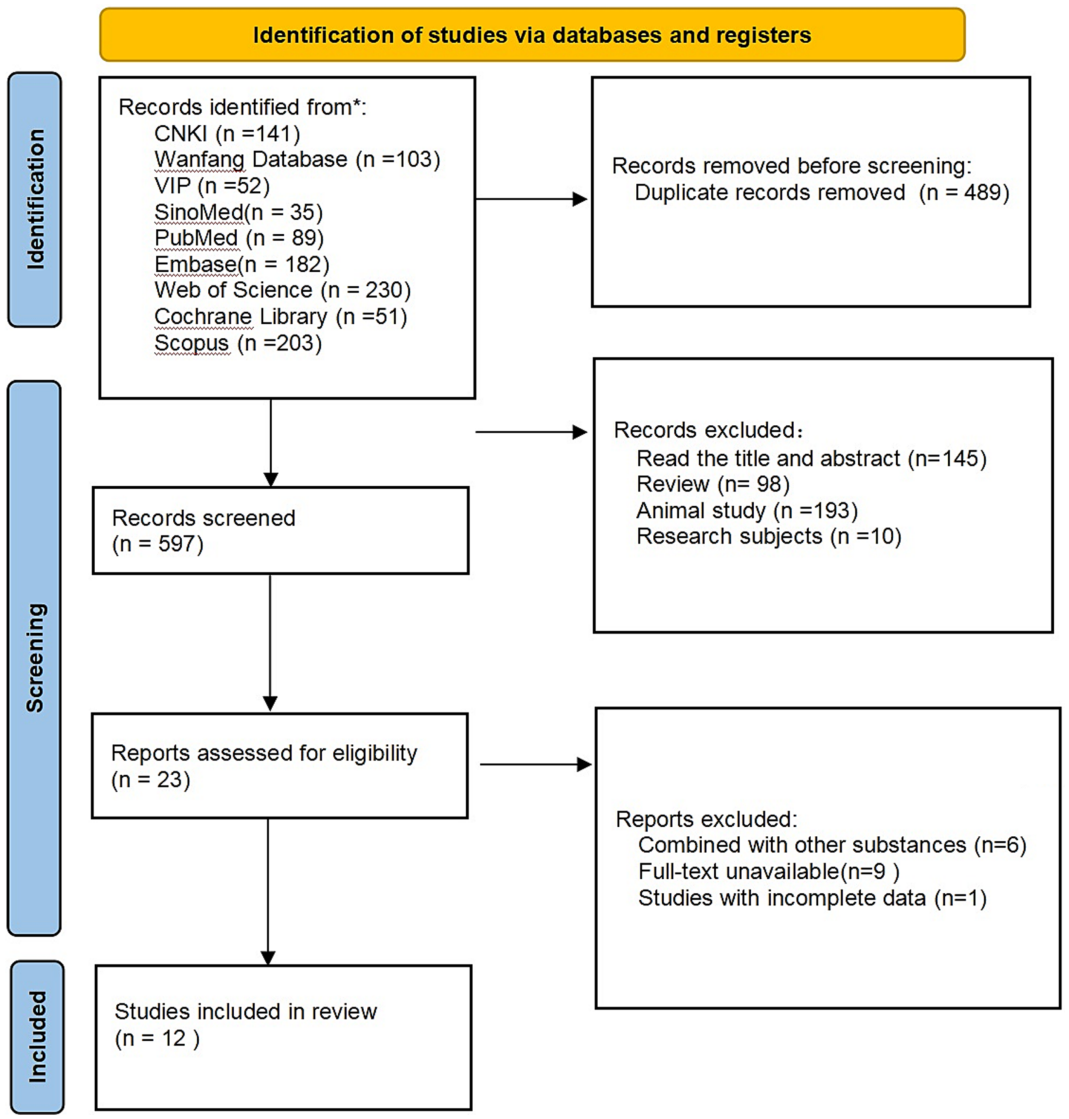
Literature screening process.

A total of 1,086 relevant literature was obtained from the preliminary search database, and after excluding 489 duplicate literatures, 597 literatures remained. After the titles and abstracts were assessed, 564 studies that failed to meet the inclusion criteria were eliminated. Yielding 23 potentially eligible publications. Upon further evaluation of the full texts, 11 publications were excluded. Consequently, a total of 12 publications were incorporated into the final analysis. The details of the literature selection process and outcomes are presented in Figure 1.

### Basic characteristics of the included studies

This study included a total of 12 trials involving 731 patients, comprising 371 in the propolis experimental group and 360 in the placebo control group. All included studies provided descriptions of the baseline characteristics for both groups and reported the outcome measures, ensuring comparability. The basic information of the included studies is presented in Tables 1, 2.

**Table 1:** Basic characteristics of the included literature.

First author, publication year	Country	Place of origin	Sample size (T/C)	Sex (male/female) (T/C)	Intervention (daily dose)	Control intervention	Duration	Outcomes	Outcomes detail
Liting Zhao, 2016 (35)	China	Brazilian propolis	65 (33/32)	T:18/15 C:14/18	Capsules 900 mg/day	Placebo capsules	18 weeks	②:e,f ③:h,j ④:k,l	Intervention significantly decreased TNF-α but increased IL-6, with no significant changes in HbA1c, SOD, or MDA levels.
Mehrnoosh Zakerkish, 2019 (30)	Iran	Iranian propolis	94 (50/44)	T:17/33 C:16/28	Capsules 1,000 mg/day	Placebo capsules	90 days	①:a,b,c,d ②:e,f,g ③:h,i,j	HbA1c, HOMA- IR, CRP, and TNF-α levels were significantly decreased in the intervention group. HDL-C levels were increased.
Fatemeh Moayedi, 2023 (28)	Iran	Italian propolis	30 (15/15)	Unreported	Capsules 500 mg/day	nothing	8 weeks	①:a, b, c, ②:f ④:k,l	In the intervention group, HbA1c and lipid levels were improved, SOD increased significantly, and MDA decreased.
Fatemeh Afsharpour, 2022 (26)	Iran	Iranian propolis	60 (30/30)	Unreported	Capsules 1,500 mg/day	Placebo capsules	2 months	①: a, b, c, d, ③:h,i	The intervention resulted in significant improvement in serum lipids with concomitant reduction in mean CRP and TNF-α levels.
Weina Gao, 2018 (34)	China	Chinese propolis	61 (30/31)	T:11/20 C:14/16	Capsules 900 mg/day	nothing	18 weeks	②:f ③:h,j ④:k	Intervention significantly elevated serum IL-6 without altering intergroup HbA1c levels.
Takuya Fukuda, 2015 (29)	Japan	Brazilian propolis	80 (41/39)	T:27/14 C:19/20	Tablets 226.8 mg/day	Placebo tablets	8 weeks	①: a, b, c, d, ②:e,f,g ③:h,i,j	There were no significant differences in blood lipid, blood glucose, and inflammation indicators between the two groups
Hesham El-Sharkawy, 2016 (40)	Egypt	Egyptian propolis	50 (24/26)	T:16/8 C:17/9	Capsules 400 mg/day	Placebo capsules	6 months	②:e,f	HbA1c and FBS levels were significantly decreased in the propolis group
Paola D. Ochoa-Morales, 2022 (85)	Mexico	American propolis	24 (12/12)	T:8/4 C:5/7	Capsules600 mg/day	Placebo capsules	12 weeks	①: a, b, c, d, ②:e,f	Propolis significantly lowered HbA1c and FBS levels, while lipid levels remained unchanged.
Mojgan Yousefi, 2023 (41)	Iran	Iranian propolis	60 (30/30)	Unreported	Capsules 1,500 mg/day	Placebo capsules	8 weeks	②:e,g ③:j	Propolis improved blood glucose status, reduced insulin resistance, and inflammation.
Wang kun fang, 2024 (42)	China	Chinese propolis	90 (45/45)	T:20/25 C:18/27	Tablets 600 mg/day	nothing	14 days	②:e ③:i,j	FBS, CRP, and IL-6 levels were significantly decreased in the propolis group
Nazli Samadi, 2017 (27)	Iran	Iranian propolis	57 (30/27)	T:13/17 C:16/11	Tablets 900 mg/day	Placebo tablets	12 weeks	①: a, b, c, d, ②:e,f,g	In the intervention group, FBS and HbA1c decreased significantly, while HDL and TG levels improved but not significantly.
Fatemeh Afsharpour, 2019 (33)	Iran	Iranian propolis	60 (30/30)	Unreported	Capsules 1,500 mg/day	Placebo capsules	2 months	②:e,f,g ④:l	FBS, HOMA-IR, and HbA1c decreased significantly, and SOD activity increased in the intervention group

① Blood lipid: a. LDL-C (Low-density lipoprotein cholesterol); b. TC (Total cholesterol); c. HDL-C (High-density lipoprotein cholesterol); d. TG (Triglyceride); ② blood glucose index: e. FBS (Fasting blood sugar); f. HbA1c (Hemoglobin A1c); g. HOMA-IR (Insulin resistance); ③ inflammatory indicators: h. TNF-α (Tumor necrosis-factorα); i. CRP (C-reactive protein); j. IL-6(interleukin-6); ④ oxidative stress index: k. malondialdehyde (MDA); l. superoxide dismutase (SOD).

**Table 2:** The outcomes included in the literature review.

First author, year	Lipid parameters (TC/TG/LDL-C/HDL-C)	Glycemic parameters (FBS/HbA1c/HOMA-IR)	Inflammatory markers (TNF-α/CRP/IL-6)	Oxidative stress markers (MDA/SOD)
Liting Zhao, 2016 (35)			TNF-α↓, IL-6↑	SOD and MDA remained unchanged
Mehrnoosh Zakerkish, 2019 (30)	HDL-C↑	HbA1c, HOMA- IR↓	, CRP, TNF-α↓	
Fatemeh Moayedi, 2023 (28)	TC, LDL-C↓, HDL-C↑	HbA1c↓		SOD↑, MDA↓
Fatemeh Afsharpour, 2022 (26)	TC, TG, LDL-C↓, HDL-C↑		CRP, TNF-α↓	
Weina Gao, 2018 (34)		HbA1c remained unchanged	IL-6↓	
Takuya Fukuda, 2015 (29)	No changed	No changed	No changed	
Hesham El-Sharkawy, 2016 (40)		FBS, HbA1c↓		
Paola D. Ochoa-Morales, 2022 (85)	No changed	FBS, HbA1c↓		
Mojgan Yousefi, 2023 (41)		FBS, HOMA-IR↓	IL-6↓	
Wang kun fang, 2024 (42)		FBS↓	CRP, IL-6↓	
Nazli Samadi, 2017 (27)	HDL and TG levels improved, but not significantly	FBG, HbA1c↓.		
Fatemeh Afsharpour, 2019 (33)		FBS, HOMA-IR HbA1c↓		SOD↑

① Lipid Parameters: LDL-C (low-density lipoprotein cholesterol); TC (total cholesterol); HDL-C (high-density lipoprotein cholesterol); TG (triglyceride); ② Glycemic Parameters: FBS (Fasting blood sugar); HbA1c (Hemoglobin A1c); HOMA-IR (Insulin resistance); ③ Inflammatory Markers: TNF-α (tumor necrosis-factorα); CRP (C-reactive protein); IL-6 (interleukin-6); ④ Oxidative Stress Markers: malondialdehyde (MDA); superoxide dismutase (SOD).

### Methodological quality of the included studies

**Figure 2:**
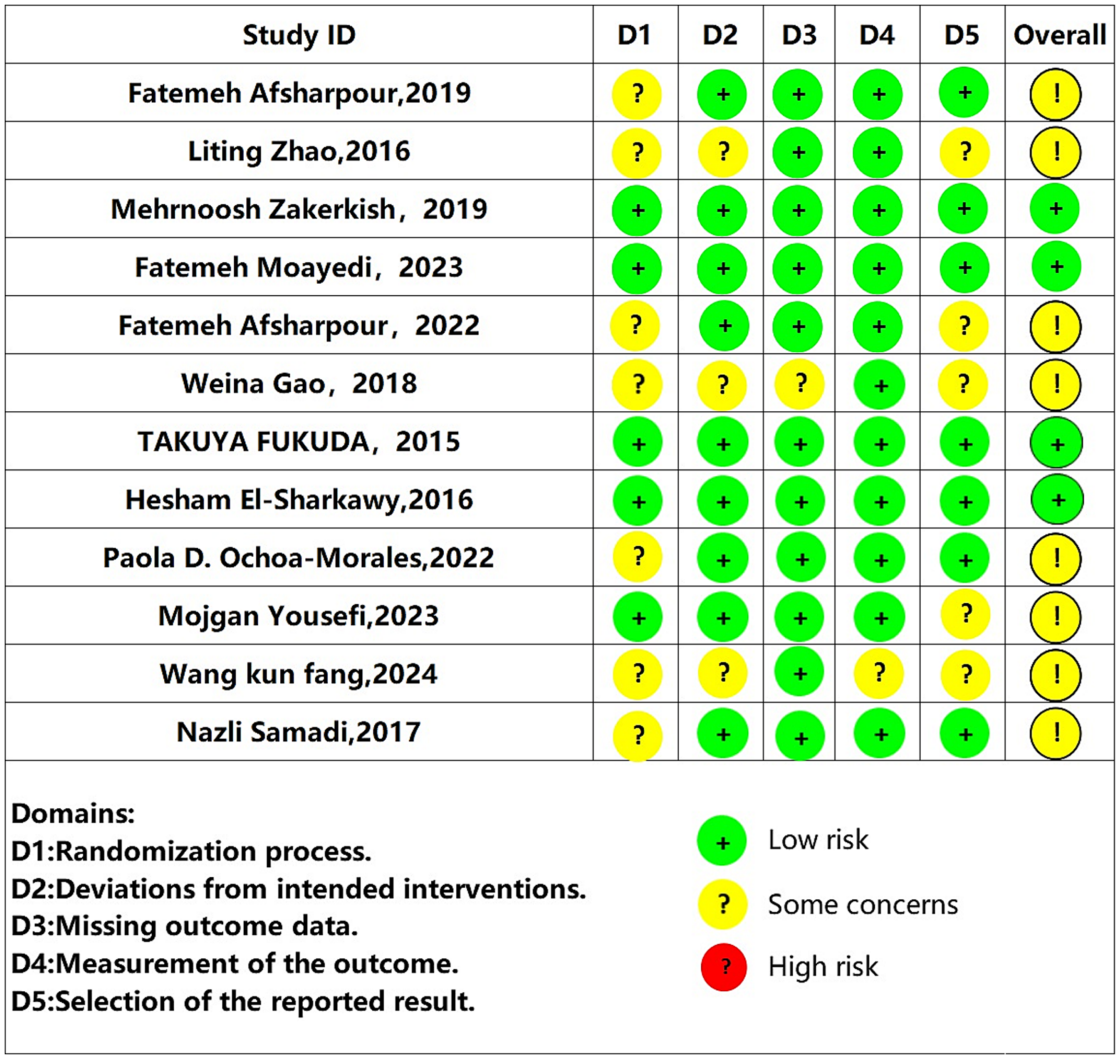
Results of the methodological quality assessment.

The quality of the methodologies employed in the 12 included studies was systematically reviewed via the Cochrane Risk of Bias Assessment Tool, version 2 (RoB 2) (39). All studies were randomized controlled trials (RCTs), but evidence of bias was identified in their randomization procedures. Notably, five studies (28–30, 40, 41) implemented detailed randomization methods with allocation concealment. The methodological quality assessment results are presented in Figure 2 and Table 3.

**Table 3:** Risk of bias summary of the included randomized controlled trials on propolis supplementation for type 2 diabetes mellitus.

Study	Randomization process	Deviations from intended interventions	Missing outcome data	Measurement of the outcome	Selection of the reported result	Overall
Fatemeh Afsharpour, 2019	S	L	L	L	L	S
Liting Zhao, 2016	S	S	L	L	S	S
Mehrnoosh Zakerkish, 2019	S	S	L	L	S	S
Fatemeh Moayedi, 2023	L	L	L	L	L	L
Fatemeh Afsharpour, 2022	L	L	L	L	L	L
Weina Gao, 2018	S	S	S	L	S	S
Takuya Fukuda, 2015	L	L	L	L	L	L
Hesham El-Sharkawy, 2016	L	L	L	L	L	L
Paola D. Ochoa-Morales, 2022	S	L	L	L	L	S
Mojgan Yousefi, 2023	L	L	L	L	S	S
Wang kun fang, 2024	S	S	L	S	S	S
Nazli Samadi, 2017	S	L	L	L	L	S

Low risk of bias; H, high risk of bias; S, Some concerns.

### Quality of evidence

The certainty of evidence for propolis supplementation’s effects on metabolic and inflammatory biomarkers was evaluated using the GRADE framework. Evidence for TG, LDL-C, HDL-C, FBS, HbA1c, and HOMA-IR was rated as low certainty, primarily due to serious risk of bias and imprecision. For TC, IL-6, CRP, and TNF-*α*, the evidence was of very low certainty, attributed to very serious imprecision (wide confidence intervals crossing the null value) alongside serious risk of bias, precluding definitive conclusions. Key downgrading factors included inadequate randomization, lack of allocation concealment, insufficient blinding, and small sample sizes. The full GRADE evidence profile is detailed in Table 4.

**Table 4:** Quality assessment.

Quality assessment							Effect	Quality	Importance
No of studies	Design	Risk of bias	Inconsistency	Indirectness	Imprecision	Other considerations	Rate (95%CI)		
TC (better indicated by lower values)									
6	Randomized trials	Serious^1^	No serious inconsistency	No serious indirectness	Very serious^3^	None	MD 0.18 lower (0.54 lower to 0.17 higher)	⊕ΟΟΟ VERY LOW	CRITICAL
TG (better indicated by lower values)									
5	Randomized trials	Serious^1^	No serious inconsistency	No serious indirectness	Serious^2^	None	MD 0.15 lower (0.3–0.01 lower)	⊕⊕ΟΟ LOW	CRITICAL
LDL-C (better indicated by lower values)									
6	Randomized trials	Serious^1^	No serious inconsistency	No serious indirectness	Serious^2^	None	MD 0.34 lower (0.42–0.26 lower)	⊕⊕ΟΟ LOW	CRITICAL
HDL-C (better indicated by lower values)									
6	Randomized trials	Serious^1^	No serious inconsistency	No serious indirectness	Serious^2^	None	MD 0.13 higher (0.1–0.16 higher)	⊕⊕ΟΟ LOW	CRITICAL
FBS (better indicated by lower values)									
9	Randomized trials	Serious^1^	No serious inconsistency	No serious indirectness	Serious^3^	None	MD 1.13 lower (2–0.27 lower)	⊕⊕ΟΟ LOW	CRITICAL
HbA1c (better indicated by lower values)									
9	Randomized trials	Serious^1^	No serious inconsistency	No serious indirectness	Serious^2^	None	MD 0.44 lower (0.78–0.11 lower)	⊕⊕ΟΟ LOW	CRITICAL
HOMA-IR (better indicated by lower values)									
5	Randomized trials	Serious^1^	No serious inconsistency	No serious indirectness	Serious^2^	None	MD 1.23 lower (1.32–1.15 lower)	⊕⊕ΟΟ LOW	IMPORTANT
CRP (better indicated by lower values)									
6	Randomized trials	Serious^1^	No serious inconsistency	No serious indirectness	Serious^2^	None	MD 1.56 lower (3.82 lower to 0.71 higher)	⊕ΟΟΟ VERY LOW	IMPORTANT
TNF-α (better indicated by lower values)									
5	Randomized trials	Serious^1^	No serious inconsistency	No serious indirectness	Very serious^3^	None	MD 2.52 lower (5.69 lower to 0.66 higher)	⊕ΟΟΟ VERY LOW	IMPORTANT
IL-6 (better indicated by lower values)									
6	Randomized trials	Serious^1^	No serious inconsistency	No serious indirectness	Very serious^3^	None	MD 0.38 lower (2.29 lower to 1.53 higher)	⊕ΟΟΟ VERY LOW	IMPORTANT

^1^The included studies were assessed as having a high risk of bias due to deficiencies in randomization, allocation concealment, and blinding. ^2^The included studies were limited by small sample sizes. ^3^The included studies were limited by small sample sizes, resulting in wide confidence intervals that indicate imprecision of effect estimates.

### Effect of propolis on blood lipids in patients with T2DM

**Figure 3:**
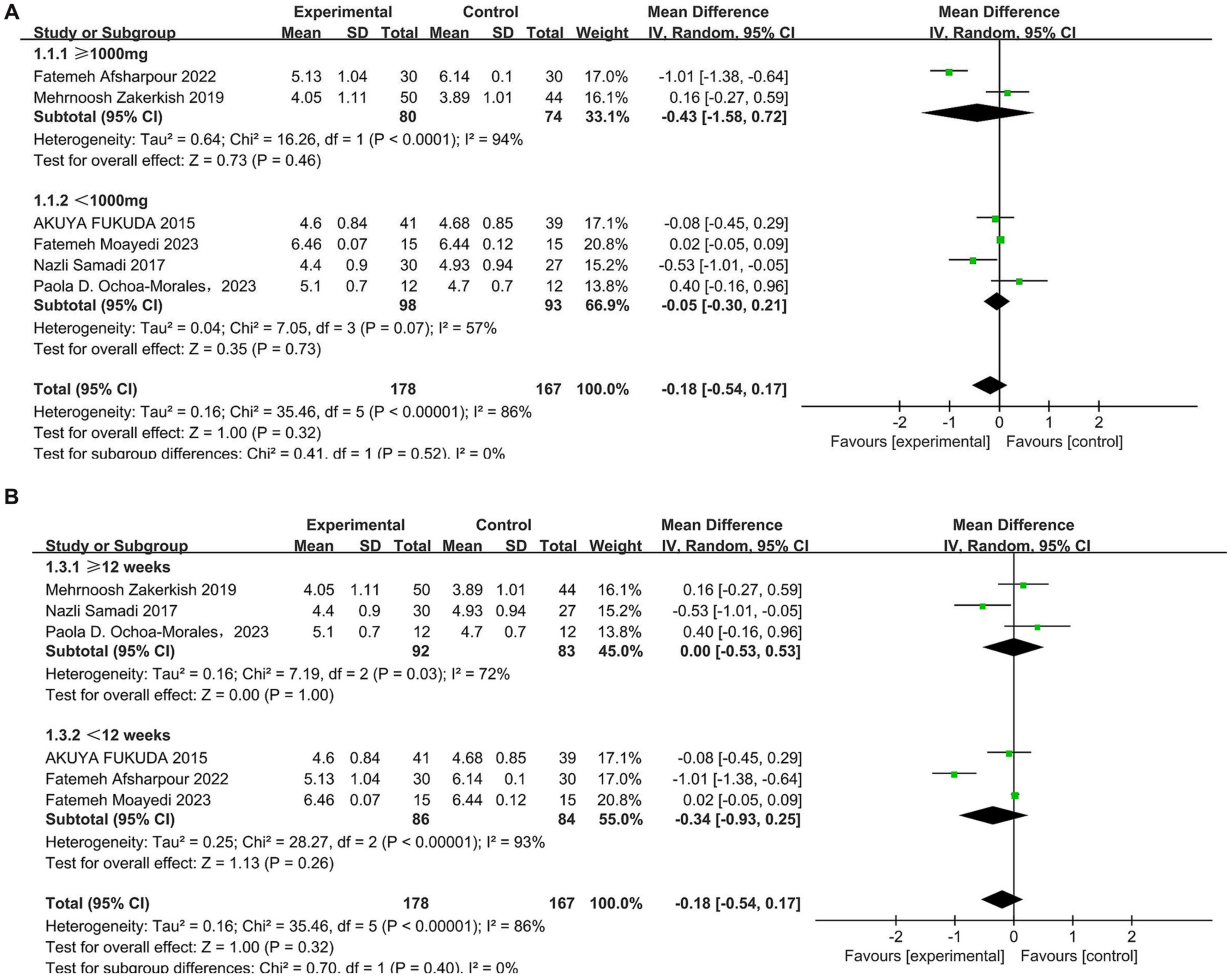
Subgroup analysis of propolis intervention on TC in T2DM patients stratified by dose **(A)** and duration **(B)**.

A total of six studies (30–34, 42) reported the effect of propolis on total cholesterol (TC) in patients with T2DM. Heterogeneity was observed among these studies (*p* < 0.00001, I^2^ = 86%). Thus, a random-effects model was employed for the analysis. The pooled results showed no significant improvement in TC levels following propolis intervention (MD = −0.18, 95% CI: −0.54–0.17, *p* = 0.32). Further subgroup analysis revealed that neither intervention dosage nor duration significantly influenced TC outcomes (Figures 3a,b and Table 5).

**Table 5:** Risk of bias summary of the included randomized controlled trials on propolis supplementation for type 2 diabetes mellitus.

Categories	Study	Pooled effect size (95% CI)	Heterogeneity (I^2^)	P-heterogeneity	*P*-value
Propolis intake on serum TC (mmol/L)					
Overall effect	6	−0.18[−0.54, 0.17]	86%	<0.0001	0.32
Intervention dose					
(mg/day)					
<1,000	4	−0.05 [−0.30, 0.21]	57	0.07	0.73
≥1,000	2	−0.43 [−1.58, 0.72]	94	<0.0001	0.46
Trial duration (week)					
<12	3	−0.34 [−0.93, 0.25]	93	<0.00001	0.26
≥12	3	0.00 [−0.53, 0.53]	72	0.03	1
Propolis intake on serum LDL-C (mmol/L)					
Overall effect	6	−0.32[−0.56, −0.08]	80%	0.0002	0.009
Trial duration (week)					
<12	3	−0.47[−0.82, −0.12]	88	0.0003	0.008
≥12	3	−0.12 [−0.39, 0.16]	34	0.22	0.40
Propolis intake on serum HDL-C (mmol/L)					
Overall effect	6	0.13 [0.10, 0.16]	0%	0.76	<0.00001
Propolis intake on serum TG (mmol/L)					
Overall effect	5	−0.15[−0.30, −0.01]	0%	0.50	0.04
Propolis intake on serum FBS (mmol/L)					
Overall effect	9	−1.13[−2.00, −0.27]	92%	<0.00001	0.01
Intervention dose					
(mg/day)					
<1,000	6	−1.16 [−2.40, 0.08]	95	<0.00001	0.07
≥1,000	3	−1.16[−1.67, −0.66]	0	0.43	<0.00001
Propolis intake on serum HOMA-IR					
Overall effect	5	−0.95 [−1.36, −0.55]	92%	<0.00001	<0.00001
Intervention dose					
(mg/day)					
<1,000	2	−0.21 [−0.52, 0.10]	0	0.54	0.18
≥1,000	3	−1.32[−1.45, −1.19]	36	0.21	<0.00001
Propolis intake on serum HbA1C (%)					
Overall effect	9	−0.44[−0.78, −0.11]	58%	0.02	0.01
Intervention dose					
(mg/day)					
<1,000	7	−0.26 [−0.59, 0.07]	39	0.13	0.12
≥1,000	2	−0.92 [−1.46, −0.39]	32	0.22	0.0007
Trial duration (week)					
<12	4	−0.24 [−0.79, 0.31]	70	0.02	0.40
≥12	5	−0.64 [−1.11, −0.17]	51	0.08	0.008
Propolis intake on serum CRP (ng/mL)					
Overall effect	3	−2.68 [−3.48, −1.89]	1%	0.37	<0.00001
Propolis intake on serum TNF-α (pg/mL)					
Overall effect	5	−2.52 [−5.69, 0.66]	70%	0.01	0.12
Intervention dose					
(mg/day)					
<1,000	3	−1.92 [−6.12, 2.27]	65	0.06	0.37
≥1,000	2	−26.67[−82.43, 29.09]	82	0.02	0.35
Trial duration (week)					
<12	4	8.21[−54.38, 70.80]	22	0.26	0.80
≥12	5	−2.96 [−8.86, 2.94]	81	0.005	0.33
Propolis intake on serum IL-6 (pg/mL)					
Overall effect	6	−0.38 [−2.29, 1.53]	95%	<0.0001	0.70
Intervention dose					
(mg/day)					
<1,000	4	−0.03 [−2.40, 2.34]	97	<0.00001	0.98
≥1,000	2	−1.32[−2.34, −0.31]	0	0.79	0.01

TC, total cholesterol; TG, triglyceride; LDL-C, low-density lipoprotein cholesterol. HDL-C, high-density lipoprotein cholesterol; FBS, fasting blood sugar; HbA1c, Hemoglobin A1c; HOMA-IR, Insulin resistance. TNF-α, tumor necrosis-factorα; CRP, C-reactive protein; IL-6, interleukin-6.

**Figure 4:**
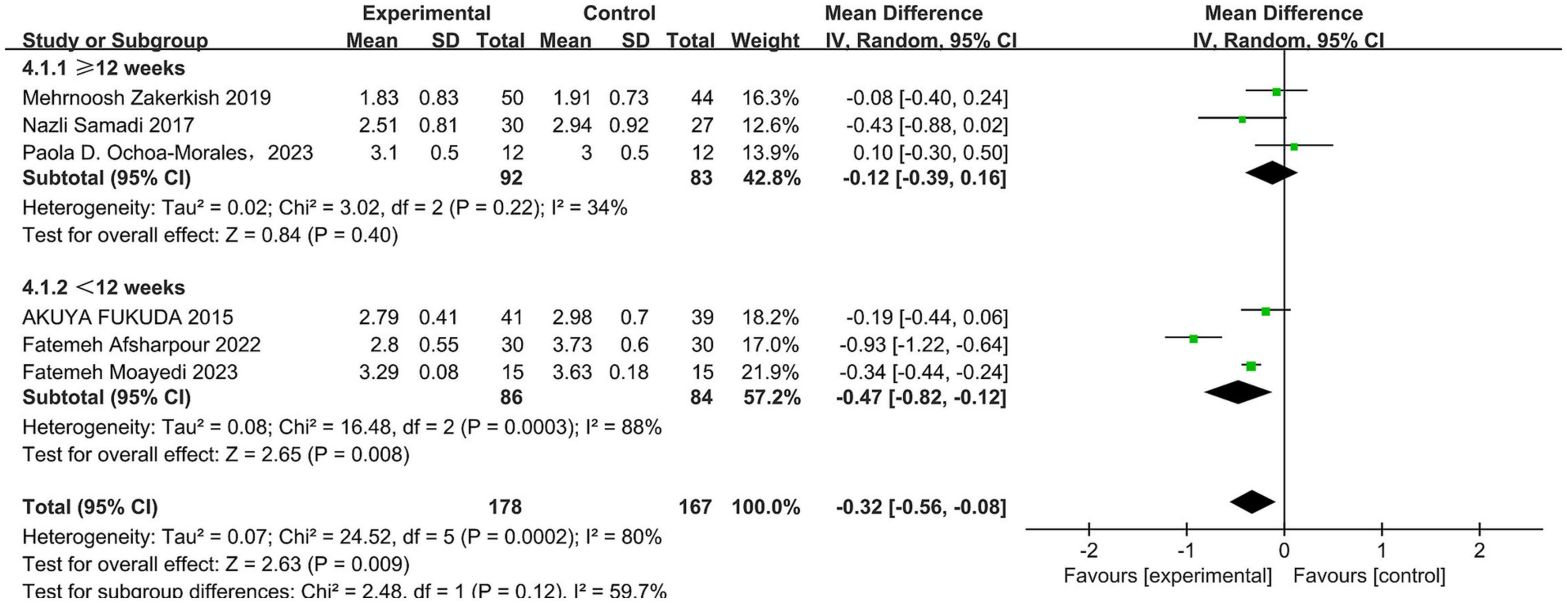
Meta-analysis results of LDL-C change in included trials.

A total of six studies (30–34, 42) reported the effect of propolis on low-density lipoprotein cholesterol (LDL-C) in patients with T2DM. Heterogeneity was observed among these studies (*p* = 0.0002, I^2^ = 80%). Thus, a random-effects model was employed for the analysis. The pooled results showed propolis significantly reduced LDL-C levels, with statistical significance (MD = −0.32, 95% CI: −0.56 to −0.08, *p* = 0.009). Subgroup analysis revealed that when the intervention duration was less than 12 weeks, propolis significantly lowered LDL-C levels in T2DM patients (MD = −0.47, 95% CI: −0.82 to −0.12, *p* = 0.0008) (see Figure 4 and Table 5).

**Figure 5:**
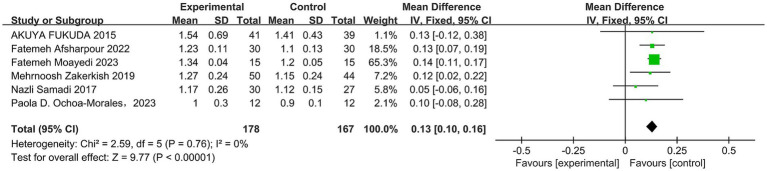
Meta-analysis results of HDL-C change in included trials.

A total of six studies (30–34, 42) reported the effect of propolis on high-density lipoprotein cholesterol (HDL-C) in T2DM patients, and there was no significant heterogeneity among the studies (*p* = 0.76, I^2^ = 0%). A fixed effect model was used, and the results revealed that propolis could improve HDL-C levels in T2DM patients, with the observed difference reaching statistical significance (MD = 0.13, 95% CI: 0.10–0.16, *p* < 0.00001) (see Figure 5 and Table 5).

**Figure 6:**
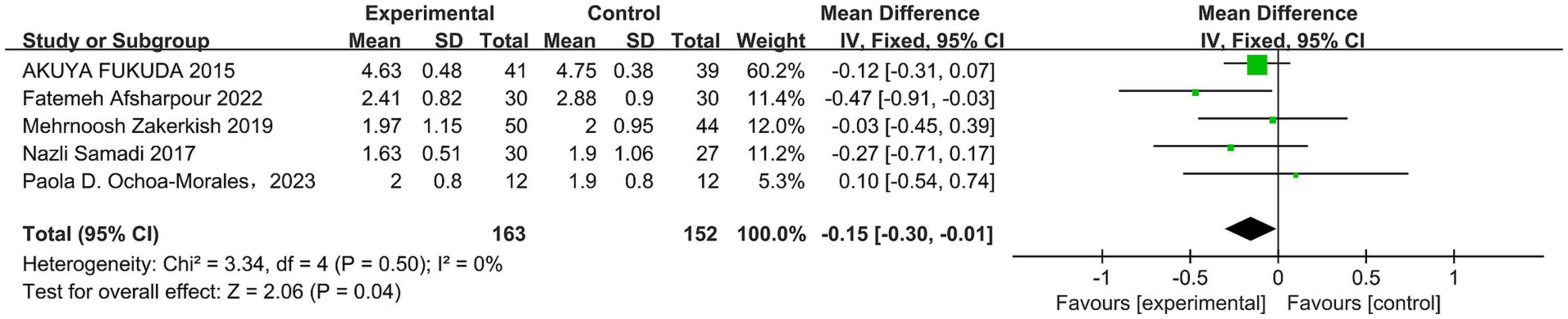
Meta-analysis results of TG change in included trials.

A total of five studies (30, 31, 33, 34, 42) reported the effect of propolis on triglyceride (TG) levels in T2DM patients, and there was no significant heterogeneity among the studies (*p* = 0.50, I^2^ = 0%). The differences were statistically significant when a fixed effects model was used (MD = −0.15, 95% CI: −0.30 to −0.01, *p* = 0.04) (see Figure 6 and Table 5).

### Effect of propolis on blood glucose in T2DM patients

**Figure 7:**
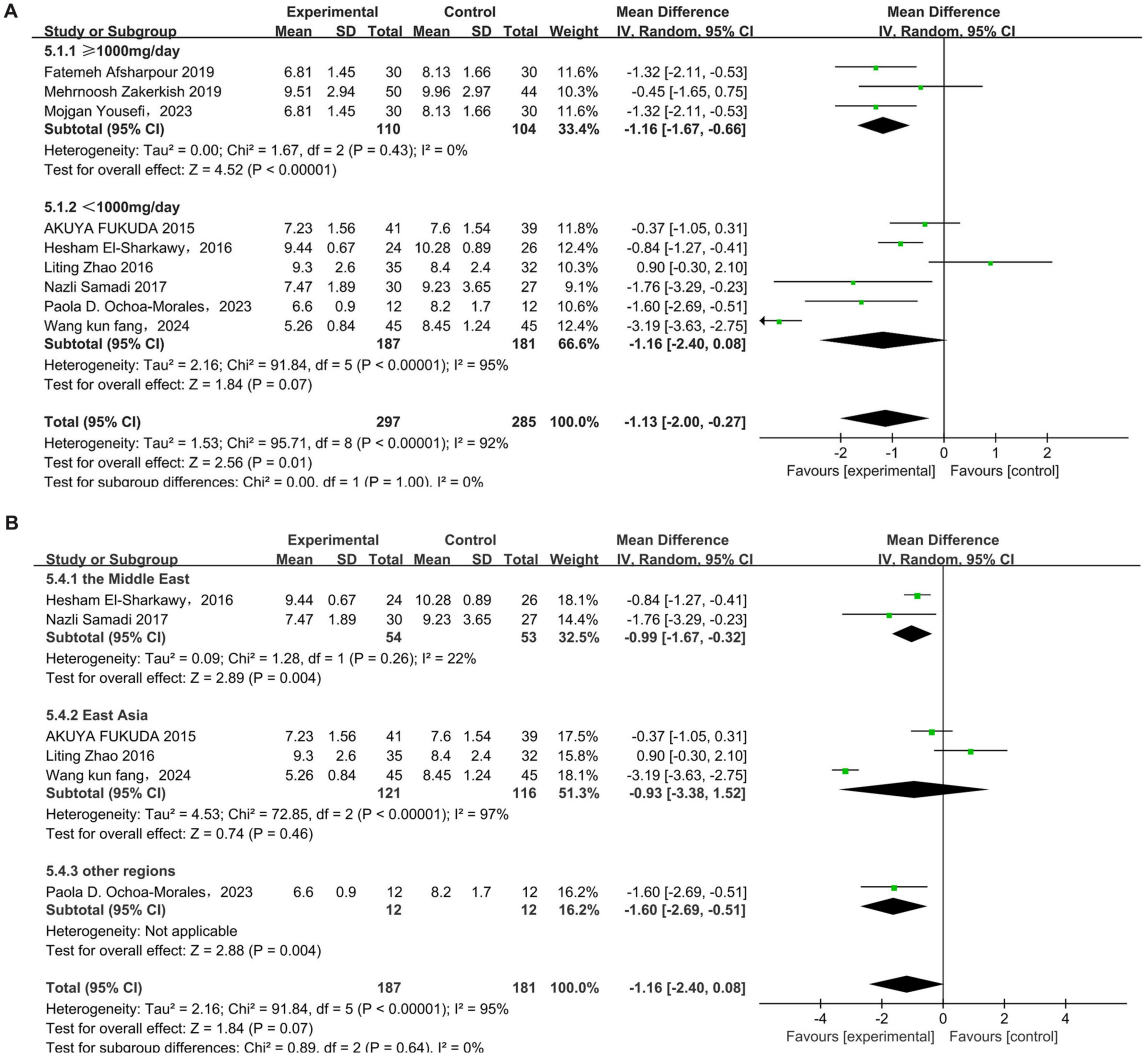
Subgroup analysis of propolis intervention on FBS in T2DM patients stratified by dose **(A)** and geographic region **(B)**.

In total, nine studies (31, 33–35, 37, 41–44) evaluated the effects of propolis on fasting blood sugar (FBS) in T2DM patients. Substantial heterogeneity was observed (*p* < 0.00001, I^2^ = 92%). Thus, a random-effects model was used for the meta-analysis. The results indicated that propolis significantly reduced FBS levels in T2DM patients (MD = −1.13, 95% CI: −2.0 to −0.27, *p* = 0.01). Subgroup analysis revealed a dose-dependent effect: a significant reduction in FBS was observed at doses ≥ 1,000 mg/day (MD = −1.16, 95% CI: −1.67 to −0.66, *p* < 0.00001). To further explore sources of heterogeneity, a subgroup analysis was performed for studies using doses <1,000 mg/day, stratified by geographic region. Among studies conducted in the Middle East, which showed low heterogeneity (I^2^ = 22%), propolis significantly improved FBS (MD = −0.99, 95% CI: −1.67 to −0.32, *p* = 0.004). In contrast, trials from East Asia showed no significant effect on FBS (MD = −0.93, 95% CI: −3.38–1.52, *p* = 0.46) (Figures 7a,b and Table 5).

**Figure 8:**
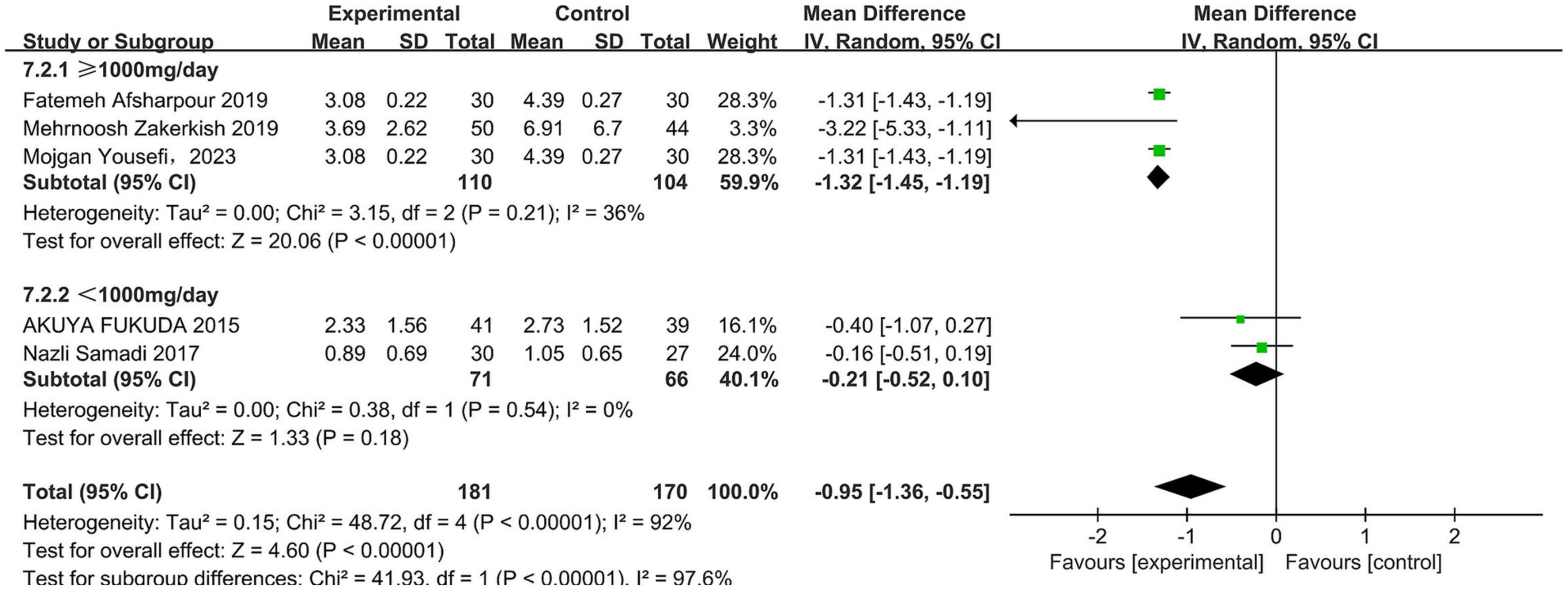
Subgroup analysis of propolis intervention on HOMA-IR in T2DM patients stratified by dose.

A total of five studies (31, 33–35, 43) reported the effect of propolis on insulin resistance (HOMA-IR) in T2DM patients, with heterogeneity among the studies (*p* < 0.00001, I^2^ = 92%), and a random-effects model was used. The results revealed that propolis can improve the level of HOMA-IR in T2DM patients, and the difference was statistically significant (MD = −0.95, 95% CI: −1.36 to −0.55, *p* < 0.00001). Subgroup analysis further showed a significant reduction only at doses ≥ 1,000 mg/day (MD = −1.32, 95% CI: −1.45 to −1.19, *p* < 0.00001) (Figure 8 and Table 5).

**Figure 9:**
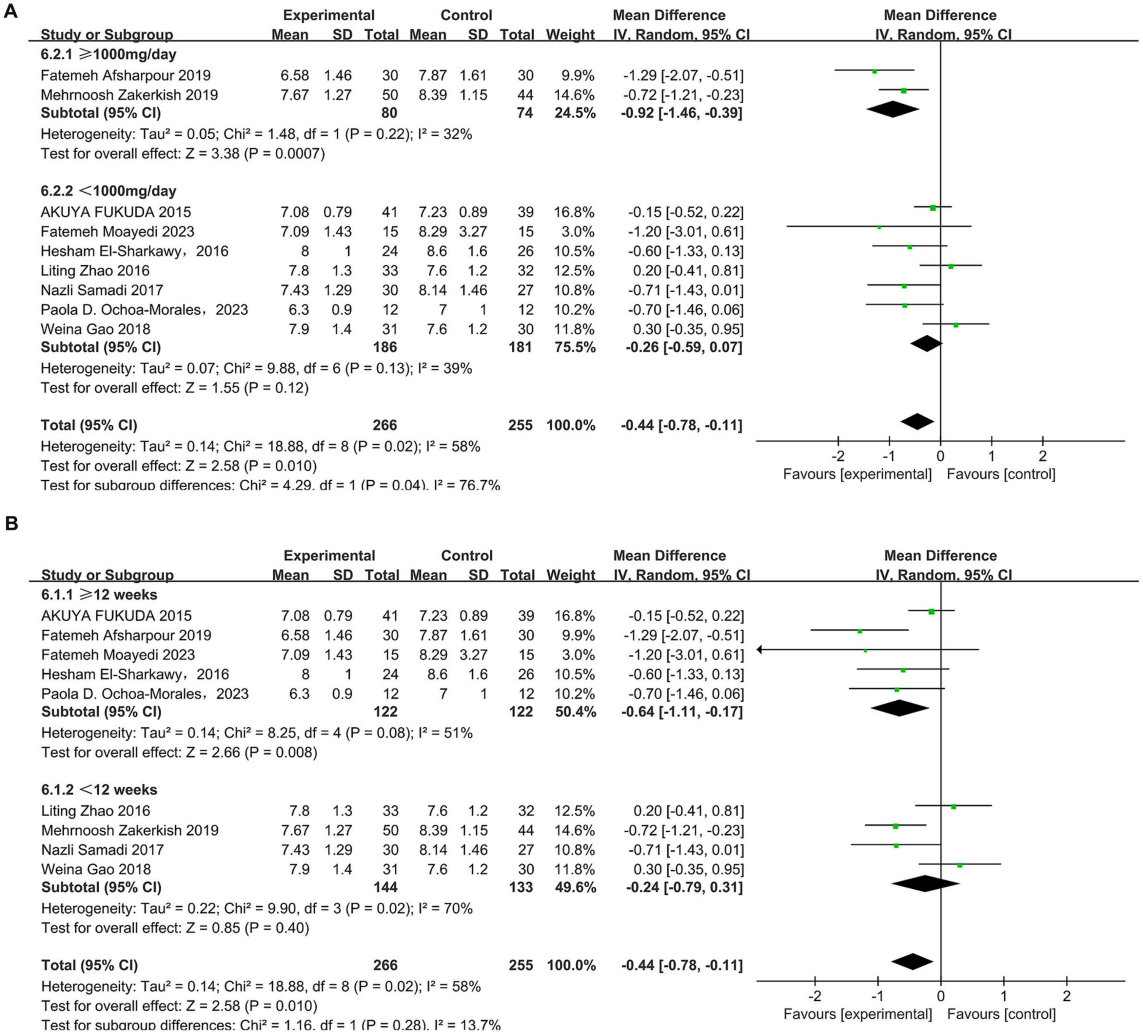
Subgroup analysis of propolis intervention on HbA1c in T2DM patients stratified by dose **(A)** and duration **(B)**.

In total, nine studies (31–37, 41, 42) reported the effect of propolis on glycosylated hemoglobin (HbA1c) in T2DM patients. Significant heterogeneity was detected across the studies (*p* = 0.02, I^2^ = 58%), and a random-effects model was used, which showed that propolis can significantly reduce the HbA1c levels (MD = −0.44, 95%CI: −0.78 to −0.11, *p* = 0.01). Subgroup analyses revealed a dose- and time-dependent effect: significant reductions were observed with doses ≥ 1,000 mg/day (MD = −0.92, 95% CI: −1.46 to −0.39, *p* = 0.0007) and durations ≥12 weeks (MD = −0.64, 95% CI: −1.11 to −0.17, *p* = 0.008) (Figure 9 and Table 5).

### Effect of propolis on inflammatory indicators in T2DM patients

**Figure 10:**
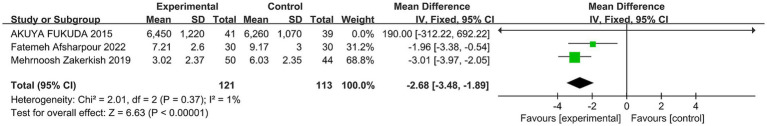
Meta-analysis results of CRP change in included trials.

A total of four studies (30, 33, 34, 44) reported the effect of propolis on C-reactive protein (CRP) in T2DM patients. One study (44) was excluded from the meta-analysis due to the inclusion of inflammatory/infective patients, which could bias results. The remaining three showed low heterogeneity (*p* = 0.37, I^2^ = 1%), so a fixed-effect model was used. Meta-analysis found propolis significantly reduced CRP (MD = −2.68, 95% CI: −3.48 to −1.89, *p* < 0.00001) (Figure 10 and Table 5).

**Figure 11:**
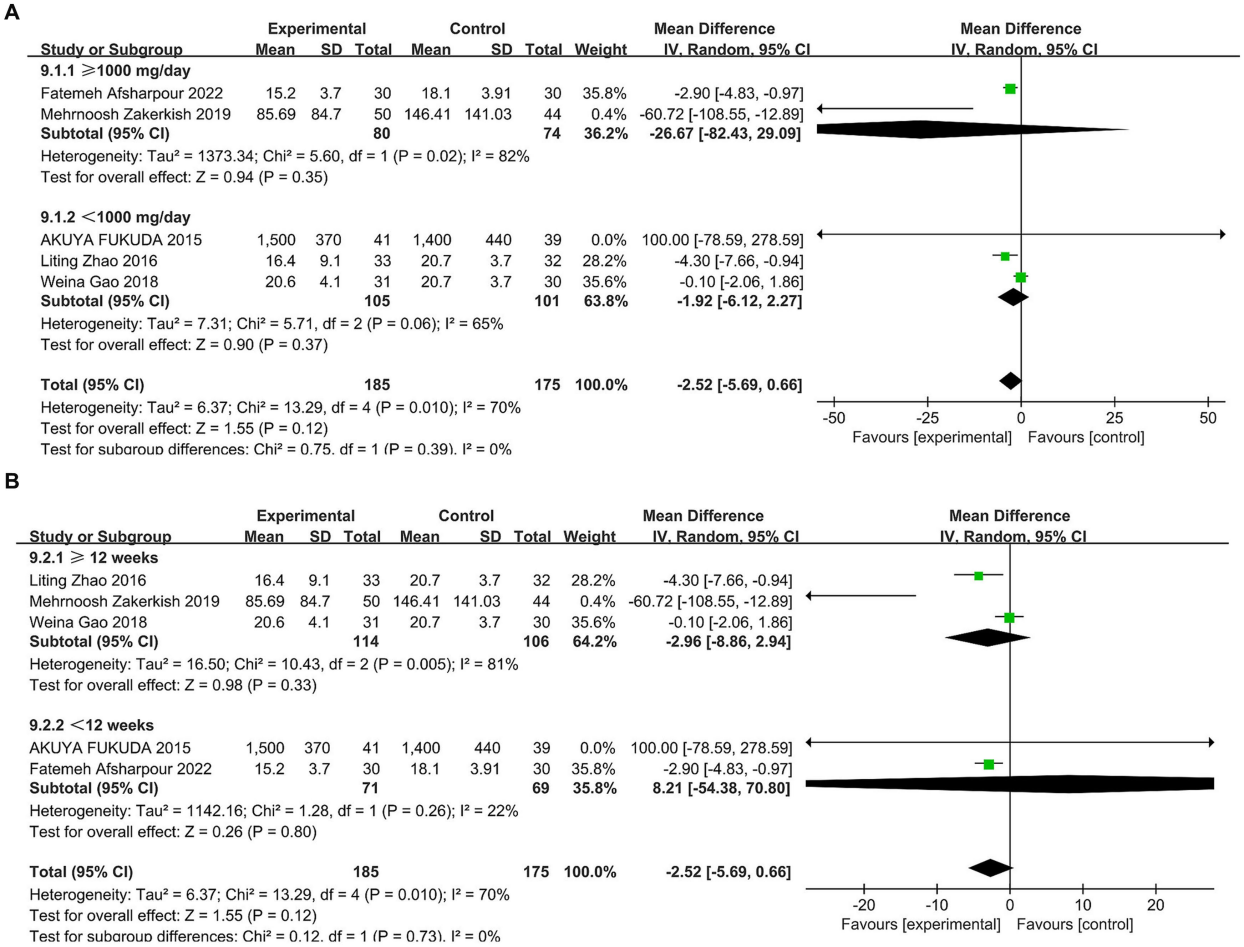
Subgroup analysis of propolis intervention on TNF-*α* in T2DM patients stratified by dose **(A)** and duration **(B)**.

In total, five studies (30, 33, 34, 36, 37) reported the effect of propolis on TNF-*α* in T2DM patients. Significant heterogeneity was detected (*p* = 0.01, I^2^ = 70%); a random effects model was used. Meta-analysis showed no statistically significant effect of propolis on TNF-*α* levels (MD = −2.52, 95% CI: −5.69–0.66, *p* = 0.12). Subgroup analyses for intervention duration and dosage also found no significant differences (Figure 11 and Table 5).

**Figure 12:**
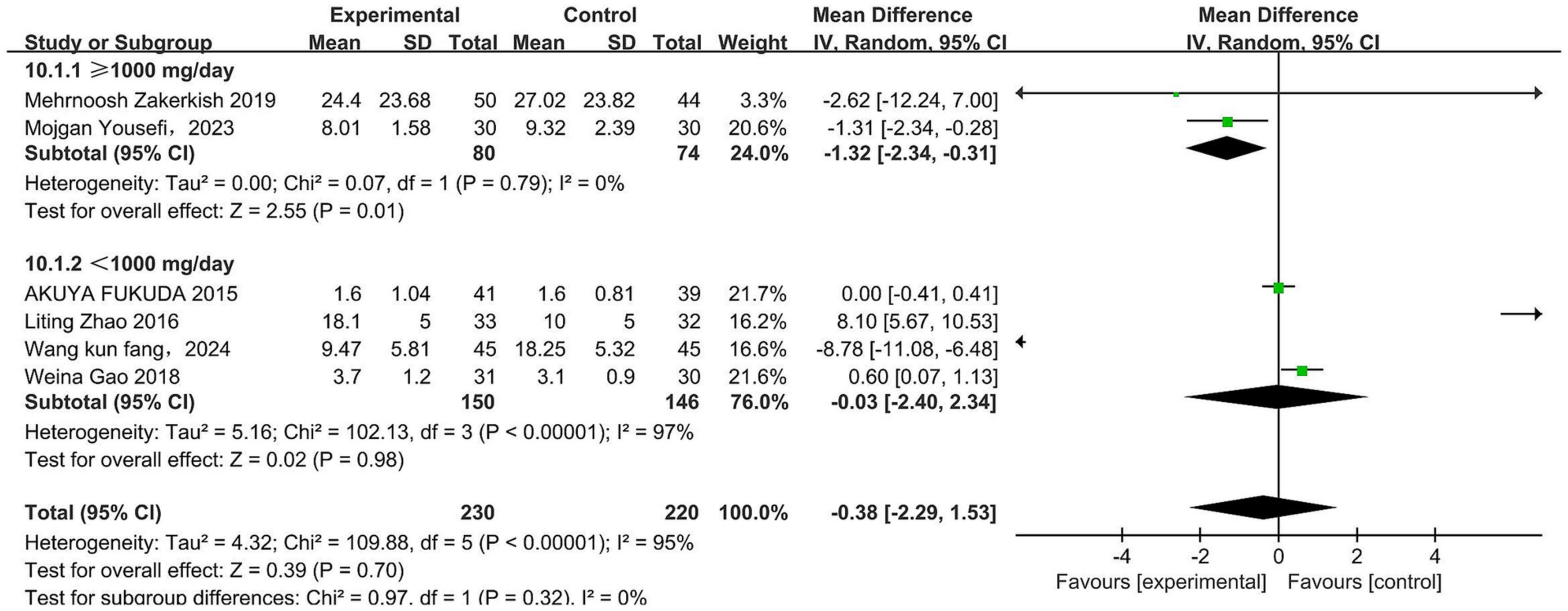
Subgroup analysis of propolis intervention on IL-6 in T2DM patients stratified by dose.

In total, six studies (33, 34, 36, 37, 43, 44) reported the effect of propolis on IL-6 in T2DM patients. Significant heterogeneity was detected (*p* < 0.00001, I^2^ = 95%). A random effects model was used, which revealed that propolis did not significantly alter IL-6 levels (MD = −0.38, 95% CI −2.29–1.53, *p* = 0.70). However, subgroup analysis showed a significant reduction at doses ≥1,000 mg/day (MD = −1.32, 95% CI −2.34 to −0.31, *p* = 0.01) (Figure 12 and Table 5).

### Effect of propolis on the oxidative stress status of T2DM patients

A total of two studies (36, 37) found no effect of propolis on MDA levels in T2DM patients. Three studies (35–37) examined SOD levels, but unit differences precluded direct comparison. One study (35) reported a significant increase in SOD post-intervention, whereas the other two (36, 37) found no significant change.

### Sensitivity analysis

Sensitivity analysis by sequential exclusion revealed stable pooled effects for TC, TG, HDL-C, LDL-C, FBS, HbA1c, HOMA-IR, CRP, and TNF-α, with confidence intervals remaining above the clinical significance threshold, confirming high robustness. However, the result for IL-6 demonstrated marked sensitivity. The initial meta-analysis, including all studies, showed no significant effect of propolis on IL-6 levels. However, after the removal of the study by Zhao et al. (37), the pooled effect became statistically significant (MD = −1.84, 95% CI −3.53 to −0.15, *p* = 0.03), suggesting a potential role of propolis in reducing IL-6. This study was identified as a key source of heterogeneity that might have masked the anti-inflammatory effect of propolis. Nevertheless, considerable heterogeneity persisted among the remaining studies (I^2^ = 94%), which precludes firm conclusions regarding its effect on IL-6.

## Discussion

This meta-analysis of 12 randomized controlled trials (RCTs) comprising 731 participants demonstrates that propolis supplementation significantly improves lipid profiles [low-density lipoprotein cholesterol (LDL-C), triglycerides (TG), high-density lipoprotein cholesterol (HDL-C)], glycemic control [fasting blood sugar (FBS), insulin resistance (HOMA-IR), hemoglobin (HbA1c)], and inflammation [C-reactive protein (CRP)] in patients with T2DM. However, current evidence is insufficient to confirm a significant effect of propolis on oxidative stress markers. This finding highlights an important direction for future research and warrants further in-depth investigation.

The present study demonstrated a downward trend in FBS, HOMA-IR, and HbA1c levels following propolis intervention, which is partially consistent with the conclusions of Karimian et al. (28). A key distinction, however, is that their research failed to detect a significant change in HOMA-IR and did not investigate the influence of dosage and intervention duration. This study not only revealed a significant reduction in HOMA-IR but also identified a dose-dependent effect, with the most pronounced improvements observed at dosages of 1,000 mg/day or higher. Similarly, while Hallajzadeh et al. (25) reported benefits for glycemic and inflammatory markers, they found no improvement in lipids and did not analyze dosage. In contrast, this study demonstrated concurrent improvements in lipid profiles, glycemic control, and inflammatory markers, and clarified the modulatory roles of both dosage and duration. Unlike previous meta-analyses (25–28), this study provides a more comprehensive evaluation by including oxidative stress and inflammatory markers. Subgroup analyses revealed that propolis at ≥1,000 mg/day significantly improved FBS, HOMA-IR, HbA1c, and IL-6 levels, with particularly pronounced HbA1c improvement at ≥12 weeks. These findings support the use of propolis as an adjunctive therapy in T2DM management.

### Effects of propolis on blood lipids in T2DM patients

This meta-analysis demonstrated that propolis supplementation significantly reduced LDL-C and TG while increasing HDL-C, but had no significant effect on TC. Compared with previous meta-analyses, the results of the present study exhibit certain discrepancies. For instance, Salehi-Sahlabadi et al. (45) reported that propolis significantly reduced TG and increased HDL-C, but had no effect on LDL-C. In contrast, two other meta-analyses (25, 46) reported no significant effects of propolis on any lipid parameters. This discrepancy is likely attributable to the differing study populations; this study was limited to patients with T2DM, whereas prior studies included non-diabetic individuals.

Currently, the mechanisms of how propolis regulates lipids are not fully clear. Lipid peroxidation, a key outcome of oxidative stress, may be mitigated by propolis flavonoids, which protect lipids from oxidative damage through multiple pathways (47). Propolis can promote the expression of the ABCA1 and ABCG1 genes, promote reverse cholesterol transport, and stimulate HDL particle formation, thereby increasing HDL-C levels (48). Study (49) indicated that upregulation of ABCA1 may be a crucial way to improve HDL-C. Propolis contains polyphenols that inhibit intestinal cholesterol absorption, reduce ox-LDL, and downregulate CD36 receptor expression, thereby decreasing macrophage uptake of ox-LDL and suppressing atherosclerotic plaque formation (13, 48). Furthermore, propolis activates PPARα in the liver to balance lipid metabolism (50), and its active component, caffeic acid phenethyl ester (CAPE), can upregulate PPAR*α* and downregulate PPARγ to relieve fat accumulation and metabolic disorders (51).

### Effect of propolis on blood glucose in T2DM patients

This meta-analysis demonstrates that propolis supplementation significantly improves glycemic control in patients with T2DM. Subgroup analyses revealed that intervention dosage and duration are critical effect modifiers. Significant reductions in FBS, HOMA-IR, and HbA1c were observed only at dosages ≥1,000 mg/day, while a clinically meaningful HbA1c reduction required an intervention duration of ≥12 weeks. Given that elevated HbA1c is a primary risk factor for diabetic microvascular complications (52, 53), the observed HbA1c-lowering effect suggests that propolis, as an adjunctive therapy, holds potential for improving long-term patient prognosis. One study (25) observed reductions in FBS and HbA1c levels following propolis supplementation, but no improvement in HOMA-IR, which is inconsistent with our findings. This discrepancy may be attributed to the heterogeneity in metabolic characteristics of the study populations. Propolis enhances glucose uptake by increasing insulin sensitivity in skeletal muscle cells, boosting GLUT4 activity, and activating the PI3K and AMPK pathways (54). It may also stimulate insulin secretion or sensitivity and inhibit intestinal *α*-glucosidase to slow carbohydrate digestion (55, 56). Compounds like galangin and pinocembrin modulate glucose metabolism in IR-HepG2 cells, improving insulin resistance (57), while total flavonoids enhance HK and PK activity, promoting glucose absorption and glycogen synthesis (58, 59).

### Effect of propolis on inflammatory indicators in T2DM patients

This meta-analysis found that propolis supplementation significantly lowers CRP levels in T2DM patients, but showed no significant overall effect on IL-6 or TNF-*α*. Notably, subgroup analysis revealed a clear dose−response relationship: IL-6 levels were significantly reduced at propolis doses ≥1,000 mg/day. Compared with previous meta-analyses (25, 60, 61), these findings are partially consistent for CRP but differ for IL-6 and TNF-α. Specifically, this study found that higher-dose propolis significantly reduced IL-6, aligning with the findings of Gholami et al. (61). However, no significant change in TNF-α levels was observed in this analysis.

Several factors may account for these discrepancies. First, the present analysis was strictly limited to T2DM patients, whereas prior studies (25, 60, 61) included non-diabetic individuals and healthy participants. As noted in study (61), the anti-inflammatory effects of propolis appear to be population-specific, with more pronounced reductions in IL-6 and TNF-α observed in Asian cohorts compared to American ones—a difference potentially attributable to geographical variations in propolis composition. This notion is supported by research (62) indicating that although both Chinese and Brazilian propolis possess anti-inflammatory properties, they differ significantly in the content of key active compounds, such as total flavonoids. Furthermore, the analysis of TNF-α in this study included only five trials, rendering it underpowered compared to the meta-analysis by Gholami et al. (61), which pooled data from 13 studies. Therefore, these results should be interpreted with caution and warrant further validation in future high-quality primary studies.

The anti-inflammatory effects of propolis are attributed to the synergistic regulation of multiple signaling pathways. A key mechanism involves the inhibition of inducible nitric oxide synthase (iNOS) by propolis, which reduces excess nitric oxide (NO) production and mitigates oxidative/nitrosative stress and subsequent tissue damage (13). The principal component, caffeic acid phenethyl ester (CAPE), is central to this action, directly suppressing iNOS transcription via NF-κB binding sites (63). CAPE, along with other flavonoids and phenolic acids, also downregulates LOX/COX-1/COX-2 in the arachidonic acid pathway, thereby blocking the synthesis of pro-inflammatory mediators like prostaglandins and leukotrienes (64, 65). Additionally, CAPE reduces pro-inflammatory cytokine mRNA levels in activated macrophages, alleviating chronic inflammation (66).

### Effect of propolis on oxidative stress in T2DM patients

The limited number of studies on oxidative stress markers prevents definitive conclusions regarding propolis’s antioxidant effects. Existing evidence remains inconsistent, with one systematic review (25) reporting no benefit, while others (27, 67) suggest positive effects. This heterogeneity may be largely attributed to variations in dosage and methodology. Subgroup analyses from previous trials (27, 67) indicated that propolis supplementation ≥1,000 mg/day significantly reduces MDA and increases SOD activity, whereas lower doses exhibit no effect, thereby highlighting dosage as a critical moderating factor. Methodological variations further complicate the comparability of results. Although all included studies (35–37) employed the thiobarbituric acid (TBA) colorimetric assay to quantify MDA, this method is known to lack specificity. The TBA reagent reacts non-specifically with other serum aldehydes, potentially leading to a systematic overestimation of MDA concentrations (68). Therefore, future studies should prioritize the standardization of more specific detection methods to enhance the reliability and comparability of research findings.

Phenolic compounds, established as the primary active and non-nutritive constituents of propolis, exhibit inherent antioxidant properties (13). These compounds mitigate oxidative stress through multiple mechanisms: they inhibit ROS-generating enzymes (e.g., phospholipase A2), scavenge free radicals, and enhance the overall antioxidant capacity (25, 47). Consequently, propolis supplementation leads to reduced MDA levels and elevated activity of antioxidant enzymes, including SOD, catalase (CAT), and glutathione peroxidase (GSH-Px), thereby alleviating oxidative stress (55, 69). Furthermore, flavonoids in propolis activate the antioxidant regulator Nrf2, bolstering cellular defenses (70, 71). Polyphenolic components such as CAPE contribute to ROS reduction by inhibiting the NF-κB pathway, which aids in protecting endothelial function (72), and by suppressing the PI3K/Akt/mTOR pathway to downregulate LOX-1 and p38 MAPK, thereby attenuating oxidative damage (73).

In recent years, non-nutrient bioactive compounds have garnered significant attention for their potential in preventing and managing diabetes (74). These compounds, which are prevalent in plant-based foods and herbs, are structurally distinct from traditional nutrients and are typically soluble in water or ethanol (75). A research team (75) proposed the “theoretical model of family nurse diet therapy,” emphasizing that polyphenols and flavonoids act synergistically to prevent and treat chronic diseases via anti-inflammatory, antioxidant, and metabolic regulatory pathways. Supporting this, additional research (70) confirmed that diets rich in polyphenolic non-nutrients can modulate metabolism and ameliorate oxidative stress, thereby helping prevent hyperlipidemia.

This theoretical framework underpins the clinical application of propolis. As a natural product abundant in polyphenols and flavonoids, propolis has generated considerable interest owing to its notable antioxidant (76), anti-inflammatory (77), anticancer (78), and antibacterial properties (79). Its efficacy stems from the synergistic interactions among its non-nutritive components, such as flavonoids and phenolic acids (73), which align closely with the core principles of the “theoretical model of family nurse diet therapy.” This alignment not only strengthens the rationale for using propolis clinically but also underscores the potential of non-nutrient components in developing natural therapeutics for diabetes.

Regarding safety, propolis, as a resinous substance, exhibits a relatively low incidence of allergic reactions. A large-scale study involving 2,007 cases reported that only 3.8% of participants experienced allergic symptoms (80). In the present analysis, two studies (36, 37) documented allergic events, leading to the withdrawal of six participants due to propolis-related allergies. The primary allergens identified are caffeic acid and its esters (81). Fortunately, bacterial biotransformation techniques have been developed to effectively remove these allergenic compounds (82), indicating that advances in processing technology may further enhance the safety profile of edible propolis products.

### Practical implications

Propolis, a natural product abundant in non-nutritive bioactive components, demonstrates potential for improving glycemic control, lipid profiles, and inflammatory markers in T2DM patients. Notably, elevated CRP levels constitute an independent risk factor for cardiovascular mortality, irrespective of diabetic status (83). The significant reduction in CRP levels associated with propolis supplementation suggests its promise as a novel adjunctive strategy for the prevention and management of both diabetes and cardiovascular disease.

This study indicates that the effects of propolis are dose- and time-dependent, with superior outcomes observed at higher doses (≥1,000 mg/day) and longer intervention durations (≥12 weeks). Consequently, for patients with inadequate glycemic control, optimizing propolis dosage and treatment duration under medical supervision may enhance therapeutic efficacy. Such individualized regimens should account for patient-specific factors, including diet, physical activity, concomitant medications, and the pharmacokinetic properties of its bioactive compounds.

Although generally safe, propolis can trigger allergic reactions in susceptible individuals (80). Pre-use allergy screening and consultation with a healthcare provider are recommended. Future research should prioritize elucidating the mechanisms of action of key bioactive constituents, establishing precise dose–response relationships, and evaluating long-term safety. To improve the synthesis of future evidence, we recommend that RCTs on propolis undergo prospective registration and adopt standardized outcome sets with uniform measurement units to reduce methodological heterogeneity.

### Strengths and limitations

To clarify the comprehensive efficacy of propolis in the management of T2DM and to address the limitations of previous research, this study conducted a systematic update and in-depth analysis. We searched both Chinese and English databases and included 12 of the latest RCTs. The quality of the included studies was assessed using the Cochrane Risk of Bias tool two. This study provides a systematic and multi-faceted review of propolis intervention in T2DM, assessing its impact not only on glycemic control but also on dyslipidemia, inflammation, and oxidative stress. By situating the findings within a theoretical framework focusing on non-nutritive compounds in chronic disease management, the study offers novel mechanistic insights into the metabolic benefits of propolis. These results not only strengthen the scientific basis for incorporating propolis into diabetes care but also have practical implications for this theoretical model in the context of chronic disease management.

The study also has several limitations. Significant heterogeneity among the included studies—stemming from variations in propolis source, dosage, intervention duration, and sample size—persisted despite statistical adjustments. The feasibility of meta-analysis for oxidative stress markers and the assessment of publication bias were precluded by an insufficient number of studies. Moreover, the generalizability of this study may be limited by the geographical homogeneity of the included research, most of which originated from Iran (30–32, 34, 35). Given that the chemical composition and biological activity of propolis vary with geographical and botanical origin (84), caution should be exercised when extrapolating these findings to propolis from other regions. The applicability of these results to other populations and healthcare settings warrants further validation.

## Conclusion

This study demonstrates that propolis significantly improves lipid, glycemic, and inflammatory parameters in patients with T2DM. These metabolic benefits are enhanced at doses ≥1,000 mg/day or intervention durations ≥12 weeks. Although no significant effect on oxidative stress markers was observed (likely due to methodological limitations such as study heterogeneity and limited sample sizes), the antioxidant potential of propolis should not be disregarded. Given the limited number of studies and the inability to assess publication bias, these findings should be interpreted cautiously. Further large-scale, multicenter randomized controlled trials are needed to confirm its clinical efficacy.

## Data Availability

The original contributions presented in the study are included in the article/Supplementary material, further inquiries can be directed to the corresponding author.

## Footnotes

^1^ https://www.crd.york.ac.uk/PROSPERO/#loginpage

## References

[1] Ma CX Ma XN Guan CH Li YD Mauricio D Fu SB. Cardiovascular disease in type 2 diabetes mellitus: progress toward personalized management. Cardiovasc Diabetol. (2022) 21:74. doi: 10.1186/s12933-022-01516-6, PMID: 35568946 PMC9107726

[2] Valabhji J Kar P. Rise in type 2 diabetes shows that prevention is more important than ever. BMJ. (2023) 381:910. doi: 10.1136/bmj.p910, PMID: 37185358

[3] Home, Resources, Diabetes Diabetes and kidney disease IDF Diabetes Atlas. (2024) Available online at: https://diabetesatlas.org/atlas/diabetes-and-kidney-disease/.

[4] World Health Organization. Diabetes[EB/OL]//Diabetes. (2024). Available online at: https://www.who.int/news-room/fact-sheets/detail/diabetes.

[5] Sheu WHH Ji LN Nitiyanant W Sheu WH Baik SH Yin D . Hypoglycemia is associated with increased worry and lower quality of life among patients with type 2 diabetes treated with oral antihyperglycemic agents in the Asia-Pacific region. Diabetes Res Clin Pract. (2012) 96:141–8. doi: 10.1016/j.diabres.2011.12.027, PMID: 22265956

[6] Cheng HJ Weng SH Wu JL Yeh ST Chen HF Novida H . Long-term sulfonylurea use and impaired awareness of hypoglycemia among patients with type 2 diabetes in Taiwan. Ann Fam Med. (2024) 22:309–16. doi: 10.1370/afm.3129, PMID: 38914437 PMC11268696

[7] Bailey CJ. Metformin: therapeutic profile in the treatment of type 2 diabetes. Diabetes Obes Metab. (2024) 26:3–19. doi: 10.1111/dom.15663, PMID: 38784991

[8] Longo M Bellastella G Maiorino MI Meier JJ Esposito K Giugliano D. Diabetes and aging: from treatment goals to pharmacologic therapy. Front Endocrinol. (2019) 10:45. doi: 10.3389/fendo.2019.00045, PMID: 30833929 PMC6387929

[9] International Hypoglycaemia Study Group. Hypoglycaemia, cardiovascular disease, and mortality in diabetes: epidemiology, pathogenesis, and management. Lancet Diabetes Endocrinol. (2019) 7:385–96. doi: 10.1016/S2213-8587(18)30315-2, PMID: 30926258

[10] Mokgalaboni K Mashaba RG Phoswa WN Lebelo SL. Curcumin attenuates hyperglycemia and inflammation in type 2 diabetes mellitus: quantitative analysis of randomized controlled trial. Nutrients. (2024) 16:4177. doi: 10.3390/nu16234177 39683570 PMC11644433

[11] Mokgalaboni K Lebelo SL Modjadji P Ghaffary S. Okra ameliorates hyperglycaemia in pre-diabetic and type 2 diabetic patients: a systematic review and meta-analysis of the clinical evidence. Front Pharmacol. (2023) 14:1132650. doi: 10.3389/fphar.2023.1132650, PMID: 37077817 PMC10107009

[12] Han SM Yuan ZL Liu JP He WL Fang C. Propolis is safe for consumption as both food and medicine. J Bee. (2004) 24, 30–1. Available online at: https://kns.cnki.net/kcms2/article/abstract?v=dTSX2bdXfeByVNM33NStHUhRvqgKF3KIckSGmfI1FjlUmf4QrWBhsWXmAJYMdUXhe0OkEaYA_RRv9GHEFGF_X3Hiy-MIlTSeV2yR-iqyQF4GP5SUcymXKSvEG4N_lPrjwVS5eqEMTpMyn9HjJfVXZlSokN50j7fnDBQj-vCGHYzsOuWsujkktA==&uniplatform=NZKPT&language=CHS

[13] Chavda VP Vuppu S Balar PC Mishra T Bezbaruah R Teli D . Propolis in the management of cardiovascular disease. Int J Biol Macromol. (2024) 266:131219. doi: 10.1016/j.ijbiomac.2024.131219 38556227

[14] Aminimoghadamfarouj N Nematollahi A. Propolis diterpenes as a remarkable bio-source for drug discovery development: a review. Int J Mol Sci. (2017) 18:1290. doi: 10.3390/ijms18061290, PMID: 28629133 PMC5486111

[15] Bankova VS Popov SS Marekov NL. Isopentenyl cinnamates from poplar buds and propolis. Phytochemistry. (1989) 28:871–3. doi: 10.1016/0031-9422(89)80133-5

[16] Sforcin JM Bankova V. Propolis: is there a potential for the development of new drugs? J Ethnopharmacol. (2011) 133:253–60. doi: 10.1016/j.jep.2010.10.032, PMID: 20970490

[17] Sforcin JM. Biological properties and therapeutic applications of propolis. Phytothe Res. (2016) 30:894–905. doi: 10.1002/ptr.5605 26988443

[18] Huang S Zhang CP Wang K Li G Hu F-L. Recent advances in the chemical composition of propolis. Molecules. (2014) 19:19610–32. doi: 10.3390/molecules191219610, PMID: 25432012 PMC6271758

[19] Rivera-Yañez N Rivera-Yañez CR Pozo-Molina G Méndez-Catalá CF Méndez-Cruz AR Nieto-Yañez O. Biomedical properties of propolis on diverse chronic diseases and its potential applications and health benefits. Nutrients. (2020) 13:78. doi: 10.3390/nu13010078, PMID: 33383693 PMC7823938

[20] Okamura T Hamaguchi M Bamba R Nakajima H Yoshimura Y Kimura T . Brazilian green propolis improves gut microbiota dysbiosis and protects against sarcopenic obesity. J Cachexia Sarcopenia Muscle. (2022) 13:3028–47. doi: 10.1002/jcsm.13076, PMID: 36162824 PMC9745478

[21] Luque-Bracho A Rosales Y Vergara-Buenaventura A. The benefits of propolis in periodontal therapy. A scoping review of preclinical and clinical studies. J Ethnopharmacol. (2023) 303:115926. doi: 10.1016/j.jep.2022.115926, PMID: 36400346

[22] Pasupuleti VR Sammugam L Ramesh N Gan SH. Honey, propolis, and royal jelly: a comprehensive review of their biological actions and health benefits. Oxidative Med Cell Longev. (2017) 2017:1259510. doi: 10.1155/2017/1259510, PMID: 28814983 PMC5549483

[23] Khalil MI Sulaiman SA. The potential role of honey and its polyphenols in preventing heart diseases: a review. Afr J Tradit Complement Altern Med. (2010) 7:315–21. doi: 10.4314/ajtcam.v7i4.56693, PMID: 21731163 PMC3005390

[24] Ruiz-Bustos P Alday E Garibay-Escobar A Sforcin JM Lipovka Y Hernandez J . Propolis: antineoplastic activity, constituents, and mechanisms of action. Curr Top Med Chem. (2023) 23:1753–64. doi: 10.2174/1568026623666230321120631, PMID: 36959133

[25] Hallajzadeh J Milajerdi A Amirani E Attari VE Maghsoudi H Mirhashemi SM. Effects of propolis supplementation on glycemic status, lipid profiles, inflammation and oxidative stress, liver enzymes, and body weight: a systematic review and meta-analysis of randomized controlled clinical trials. J Diabetes Metab Disord. (2021) 20:831–43. doi: 10.1007/s40200-020-00696-w, PMID: 34178866 PMC8212256

[26] Jalali M Ranjbar T Mosallanezhad Z Mahmoodi M Moosavian SP Ferns GA . Effect of propolis intake on serum C-reactive protein (CRP) and tumor necrosis factor-alpha (TNF-α) levels in adults: a systematic review and meta-analysis of clinical trials. Complement Ther Med. (2020) 50:102380. doi: 10.1016/j.ctim.2020.102380, PMID: 32444060

[27] Nazari-Bonab H Jamilian P Radkhah N Zarezadeh M Ebrahimi-Mameghani M. The effect of propolis supplementation in improving antioxidant status: a systematic review and meta-analysis of controlled clinical trials. Phytother Res. (2023) 37:3712–23. doi: 10.1002/ptr.7899, PMID: 37317592

[28] Karimian J Hadi A Pourmasoumi M Najafgholizadeh A Ghavami A. The efficacy of propolis on markers of glycemic control in adults with type 2 diabetes mellitus: a systematic review and meta-analysis. Phytothe Res. (2019) 33:1616–26. doi: 10.1002/ptr.6356 30950136

[29] Fuliang HU Hepburn HR Xuan H Chen M Daya S Radloff SE. Effects of propolis on blood glucose, blood lipid and free radicals in rats with diabetes mellitus. Pharmacol Res. (2005) 51:147–52. doi: 10.1016/j.phrs.2004.06.011, PMID: 15629260

[30] Afsharpour F Javadi M Hashemipour S Koushan Y Khadem Haghighian H. Changes in lipid profile, liver enzymes and inflammatory factors following oral supplementation with propolis in patients with type 2 diabetes. Clin Diabetol. (2022) 11:224–31. doi: 10.5603/DK.a2022.0033

[31] Samadi N Mozaffari-Khosravi H Rahmanian M Askarishahi M. Effects of bee propolis supplementation on glycemic control, lipid profile and insulin resistance indices in patients with type 2 diabetes: a randomized, double-blind clinical trial. J Integr Med. (2017) 15:124–34. doi: 10.1016/S2095-4964(17)60315-7, PMID: 28285617

[32] Moayedi F Taghian F Dehkordi KJ Jalali Dehkordi K Hosseini SA. Cumulative effects of exercise training and consumption of propolis on managing diabetic dyslipidemia in adult women: a single-blind, randomized, controlled trial with pre-post-intervention assessments. J Physiol Sci. (2023) 73:17. doi: 10.1186/s12576-023-00872-6, PMID: 37542207 PMC10717816

[33] Fukuda T Fukui M Tanaka M Senmaru TAKAFUMI Iwase HIROYA Yamazaki MASAHIRO . Effect of Brazilian green propolis in patients with type 2 diabetes: a double-blind randomized placebo-controlled study. Biomed Rep. (2015) 3:355–60. doi: 10.3892/br.2015.436, PMID: 26137235 PMC4467280

[34] Zakerkish M Jenabi M Zaeemzadeh N Hemmati AA Neisi N. The effect of Iranian propolis on glucose metabolism, lipid profile, insulin resistance, renal function and inflammatory biomarkers in patients with type 2 diabetes mellitus: a randomized double-blind clinical trial. Sci Rep. (2019) 9:7289. doi: 10.1038/s41598-019-43838-8, PMID: 31086222 PMC6514000

[35] Afsharpour F Javadi M Hashemipour S Koushan Y haghighian HK. Propolis supplementation improves glycemic and antioxidant status in patients with type 2 diabetes: a randomized, double-blind, placebo-controlled study. Complement Ther Med. (2019) 43:283–8. doi: 10.1016/j.ctim.2019.03.001 30935545

[36] Gao W Pu L Wei J Yao Z Wang Y Shi T . Serum antioxidant parameters are significantly increased in patients with type 2 diabetes mellitus after consumption of Chinese propolis: a randomized controlled trial based on fasting serum glucose level. Diabetes Ther. (2018) 9:101–11. doi: 10.1007/s13300-017-0341-9, PMID: 29214374 PMC5801227

[37] Zhao L Pu L Wei J Li J Wu J Xin Z . Brazilian green Propolis improves antioxidant function in patients with type 2 diabetes mellitus. Int J Environ Res Public Health. (2016) 13:498. doi: 10.3390/ijerph13050498, PMID: 27187435 PMC4881123

[38] Hesami S Hashemipour S Shiri-Shahsavar MR Koushan Y Khadem Haghighian H. Administration of Iranian Propolis attenuates oxidative stress and blood glucose in type II diabetic patients: a randomized, double-blind, placebo-controlled, clinical trial. Casp J Intern Med. (2019) 10:48–54. doi: 10.22088/cjim.10.1.48, PMID: 30858941 PMC6386327

[39] Page MJ Mckenzie JE Bossuyt PM Boutron I Hoffmann TC Mulrow CD . The PRISMA 2020 statement: an updated guideline for reporting systematic reviews. BMJ. (2021) 372:n71. doi: 10.1136/bmj.n71, PMID: 33782057 PMC8005924

[40] Harreiter J Roden M. Diabetes mellitus: definition, classification, diagnosis, screening and prevention (update 2023). Wien Klin Wochenschr. (2023) 135:7–17. doi: 10.1007/s00508-022-02122-y 37101021 PMC10133036

[41] El-Sharkawy HM Anees MM Van Dyke TE. Propolis improves periodontal status and glycemic control in patients with type 2 diabetes mellitus and chronic periodontitis: a randomized clinical trial. J Periodontol. (2016) 87:1418–26. doi: 10.1902/jop.2016.150694 27468795

[42] Ochoa-Morales PD González-Ortiz M Martínez-Abundis E Pérez-Rubio KG Patiño-Laguna ADJ. Anti-hyperglycemic effects of propolis or metformin in type 2 diabetes mellitus. Int J Vitam Nutr Res. (2023) 93:498–506. doi: 10.1024/0300-9831/a000760 35965421

[43] Yousefi M Hashemipour S Shiri-Shahsavar MR Koushan Y Haghighian HK. Reducing the inflammatory interleukins with anti-inflammatory and antioxidant effects of Propolis in patients with type 2 diabetes: double-blind, randomized controlled, clinical trial. Clin Diabetol. (2023) 12:327–35. doi: 10.5603/cd.96910

[44] Wang KF Xu SH Xie X Yu KN Lv HQ. Effects of propolis supplementation on efficacy, inflammatory factors and immune function in type 2 diabetes patients with pulmonary infection. J Transl Med. (2024) 13:264–7. Available online at: https://kns.cnki.net/kcms2/article/abstract?v=dTSX2bdXfeBF87evRF4_f8MRqO_Up5i5rIIDYwRa1CwFR6QS66my4y7-OQQykwWCWV3TEXDzSM6iMoKO0xX7zkdrHi94s4BJ0yxOdxluTL85h2n5qyG6iyNKcdtgSOImV_fJEEJNqDGBFQ57qY6cYE7t7sa2J1C8rsJ_VmIg6wYDYV27XeiYKQ==&uniplatform=NZKPT&language=CHS

[45] Salehi-Sahlabadi A Chhabra M Rahmani J Momeni A Karam G Nattagh-Eshtivani E . The effect of propolis on anthropometric indices and lipid profile: a systematic review and meta-analysis of randomized controlled trials. J Diabetes Metab Disord. (2020) 19:1835–43. doi: 10.1007/s40200-020-00604-2, PMID: 33520864 PMC7843794

[46] Gheflati A Dehnavi Z Ghannadzadeh Yazdi A Khorasanchi Z Raeisi-Dehkordi H Ranjbar G. The effects of propolis supplementation on metabolic parameters: a systematic review and meta-analysis of randomized controlled clinical trials. Avicenna J Phytomed. (2021) 11:551–65. doi: 10.22038/AJP.2021.18046 34804893 PMC8588957

[47] Kumar S Pandey AK. Chemistry and biological activities of flavonoids: an overview. TheScientificWorldJournal. (2013) 2013:162750. doi: 10.1155/2013/162750, PMID: 24470791 PMC3891543

[48] Yu Y Si Y Song G Luo T Wang J Qin S. Ethanolic extract of propolis promotes reverse cholesterol transport and the expression of ATP-binding cassette transporter A1 and G1 in mice. Lipids. (2011) 46:805–11. doi: 10.1007/s11745-011-3568-7, PMID: 21638064

[49] Daleprane JB Freitas V d S Pacheco A Rudnicki M Faine LA Dörr FA . Anti-atherogenic and anti-angiogenic activities of polyphenols from propolis. J Nutr Biochem. (2012) 23:557–66. doi: 10.1016/j.jnutbio.2011.02.012 21764281

[50] Lh C Yw C Ml C CC H CH C HW T . Taiwanese green propolis ethanol extract delays the progression of type 2 diabetes mellitus in rats treated with streptozotocin/high-fat diet. Nutrients. (2018) 10:503. doi: 10.3390/nu10040503 29670038 PMC5946288

[51] Kong L Zhang Y Feng Z Dong J Zhang H. Phenolic compounds of propolis alleviate lipid metabolism disorder. Evid Based Complementary Altern Med. (2021) 2021:7615830. doi: 10.1155/2021/7615830 PMC7914084 33688365

[52] Guo G Guan Y Chen Y Ye Y Gan Z Cao X . HbA1c and the risk of lower limb ulcers among diabetic patients: an observational and genetics study. J Diabetes Res. (2025):2025: 4744194. doi: 10.1155/jdr/4744194 PMC11972128 40190410

[53] Ling J Xie Z Chen X Ling D Luo X. Inverted U-shaped relationship between HbA1c and diabetic retinopathy in diabetic patients: a cross-sectional study. BMC Ophthalmol. (2025) 25:289. doi: 10.1186/s12886-025-04079-8, PMID: 40361041 PMC12070565

[54] Ueda M Hayashibara K Ashida H. Propolis extract promotes translocation of glucose transporter 4 and glucose uptake through both PI3K- and AMPK-dependent pathways in skeletal muscle. BioFactors. (2013) 39:457–66. doi: 10.1002/biof.1085, PMID: 23355380

[55] Mujica V Orrego R Pérez J Romero P Ovalle P Zúñiga-Hernández J . The role of propolis in oxidative stress and lipid metabolism: a randomized controlled trial. Evid Based Complement Alternat Med. (2017) 2017:4272940. doi: 10.1155/2017/4272940, PMID: 28539963 PMC5429932

[56] Wei P Ding Y Lu Q Tan J Liu R. Flavonoid components in propolis and poplar resin and their inhibitory activity on α-glucosidase. J Huazhong Agric Univ. (2018) 37:92–9. doi: 10.13300/j.cnki.hnlkxb.2018.03.014

[57] Liang XL Zhao YZ Zhang HC Peng WJ. Improvement effects of four flavonoids from propolis on insulin resistance in HepG2 cells. J Agric Sci Technol. (2017) 19:74–80. doi: 10.13304/j.nykjdb.2017.0020

[58] Yang M Sui DJ Chen WX Yang M Yu DW. Hypoglycemic mechanism of total flavonoids from propolis in STZ-induced diabetic rats. J Chin Med Mater. (2014) 37:1623–6. doi: 10.13863/j.issn1001-4454.2014.09.030 25857164

[59] Liu Y Liang X Zhang G Kong L Peng W Zhang H. Galangin and pinocembrin from Propolis ameliorate insulin resistance in HepG2 cells via regulating Akt/mTOR signaling. Evid Based Complement Alternat Med. (2018) 2018:7971842. doi: 10.1155/2018/7971842, PMID: 30420897 PMC6215570

[60] Shang H Srikanth Bhagavathula A Ali Aldhaleei W Rahmani J Karam G Rinaldi G . Effect of propolis supplementation on C-reactive protein levels and other inflammatory factors: a systematic review and meta-analysis of randomized controlled trials. J King Saud Univ Sci. (2020) 32:1694–701. doi: 10.1016/j.jksus.2020.01.003

[61] Gholami A Dinarvand N Hariri M. Propolis supplementation can reduce serum level of interleukin-6, C-reactive protein, and tumor necrosis factor-α: an updated systematic review and dose-response meta-analysis on randomized clinical trials. J Health Popul Nutr. (2024) 43:119. doi: 10.1186/s41043-024-00600-9, PMID: 39127756 PMC11316998

[62] Yuan M Yuan XJ Pineda M Liang Z-Y He J Sun S-W . A comparative study between Chinese propolis and Brazilian green propolis: metabolite profile and bioactivity. Food Funct. (2020) 11:2368–79. doi: 10.1039/c9fo02051a 32129351

[63] Li D Wang X Huang Q Li S Zhou Y Li Z. Cardioprotection of CAPE-oNO2 against myocardial ischemia/reperfusion induced ROS generation via regulating the SIRT1/eNOS/NF-κB pathway in vivo and in vitro. Redox Biol. (2018) 15:62–73. doi: 10.1016/j.redox.2017.11.023, PMID: 29220696 PMC5725281

[64] Mirzoeva OK Calder PC. The effect of propolis and its components on eicosanoid production during the inflammatory response. Prostaglandins Leukot Essent Fatty Acids. (1996) 55:441–9. doi: 10.1016/s0952-3278(96)90129-5, PMID: 9014224

[65] Funk CD. Prostaglandins and leukotrienes: advances in eicosanoid biology. Science. (2001) 294:1871–5. doi: 10.1126/science.294.5548.1871, PMID: 11729303

[66] Juman S Yasui N Ikeda K Ueda A Sakanaka M Negishi H . Caffeic acid phenethyl ester suppresses the production of pro-inflammatory cytokines in hypertrophic adipocytes through lipopolysaccharide-stimulated macrophages. Biol Pharm Bull. (2012) 35:1941–6. doi: 10.1248/bpb.b12-00317, PMID: 23123466

[67] Bahari H Shahraki Jazinaki M Aliakbarian M Rashidmayvan M Golafrouz H Rahnama I . Propolis supplementation on inflammatory and oxidative stress biomarkers in adults: a systematic review and meta-analysis of randomized controlled trials. Front Nutr. (2025) 12:1542184. doi: 10.3389/fnut.2025.1542184, PMID: 40421039 PMC12104767

[68] Papastergiadis A Mubiru E VAN Langenhove H De Meulenaer B. Malondialdehyde measurement in oxidized foods: evaluation of the spectrophotometric thiobarbituric acid reactive substances (TBARS) test in various foods. J Agric Food Chem. (2012) 60:9589–94. doi: 10.1021/jf302451c, PMID: 22950760

[69] Yangi B Cengiz Ustuner M Dincer M Ozbayer C Tekin N Ustuner D . Propolis protects endotoxin induced acute lung and liver inflammation through attenuating inflammatory responses and oxidative stress. J Med Food. (2018) 21:1096–105. doi: 10.1089/jmf.2017.0151, PMID: 29719160

[70] Lee Y Shin DH Kim JH Hong S Choi D Kim Y-J . Caffeic acid phenethyl ester-mediated Nrf2 activation and IkappaB kinase inhibition are involved in NFkappaB inhibitory effect: structural analysis for NFkappaB inhibition. Eur J Pharmacol. (2010) 643:21–8. doi: 10.1016/j.ejphar.2010.06.016 20599928

[71] Levonen AL Inkala M Heikura T Jauhiainen S Jyrkkänen H-K Kansanen E . Nrf2 gene transfer induces antioxidant enzymes and suppresses smooth muscle cell growth in vitro and reduces oxidative stress in rabbit aorta in vivo. Arterioscler Thromb Vasc Biol. (2007) 27:741–7. doi: 10.1161/01.ATV.0000258868.80079.4d, PMID: 17255530

[72] Natarajan K Singh S Burke TR Grunberger D Aggarwal BB. Caffeic acid phenethyl ester is a potent and specific inhibitor of activation of nuclear transcription factor NF-kappa B. Proc Natl Acad Sci USA. (1996) 93:9090–5. doi: 10.1073/pnas.93.17.9090, PMID: 8799159 PMC38600

[73] Chang H Yuan W Wu H Yin X Xuan H. Bioactive components and mechanisms of Chinese poplar propolis alleviates oxidized low-density lipoprotein-induced endothelial cells injury. BMC Complement Altern Med. (2018) 18:142. doi: 10.1186/s12906-018-2215-8 29724195 PMC5934819

[74] Jugran AK Rawat S Devkota HP Bhatt ID Rawal RS. Diabetes and plant-derived natural products: from ethnopharmacological approaches to their potential for modern drug discovery and development. Phytother Res. (2021) 35:223–45. doi: 10.1002/ptr.6821, PMID: 32909364

[75] Han SF Feng YQ Gao WQ. Theoretical model of non-nutrient diet therapy for prevention and treatment of chronic diseases. Chin Nurs Res. (2023) 37:565–9. doi: 10.12102/j.issn.1009-6493.2023.04.001

[76] Solorzano ER Roverso M Bogialli S Bortoli M Orian L Badocco D . Antioxidant activity of Zuccagnia-type propolis: a combined approach based on LC-HRMS analysis of bioanalytical-guided fractions and computational investigation. Food Chem. (2024) 461:140827. doi: 10.1016/j.foodchem.2024.140827, PMID: 39146684

[77] Li Z Liu Z Guo Y Gao S Tang Y Li T . Propolis alleviates acute lung injury induced by heat-inactivated methicillin-resistant *Staphylococcus aureus* via regulating inflammatory mediators, gut microbiota and serum metabolites. Nutrients. (2024) 16:1598. doi: 10.3390/nu16111598, PMID: 38892531 PMC11175110

[78] Guo Y Liu Z Wu Q Li Z Yang J Xuan H. Integration with transcriptomic and metabolomic analyses reveals the in vitro cytotoxic mechanisms of Chinese poplar propolis by triggering the glucose metabolism in human hepatocellular carcinoma cells. Nutrients. (2023) 15:4329. doi: 10.3390/nu15204329, PMID: 37892405 PMC10610315

[79] Magnavacca A Sangiovanni E Racagni G Dell'Agli M. The antiviral and immunomodulatory activities of propolis: an update and future perspectives for respiratory diseases. Med Res Rev. (2022) 42:897–945. doi: 10.1002/med.21866, PMID: 34725836 PMC9298305

[80] Jian XD. Attention to propolis allergy. J Bee. (2014) 34:47. Available online at: https://kns.cnki.net/kcms2/article/abstract?v=dTSX2bdXfeDKKTWzGxIiBrt9ExgRI_Tt_6VE6RQkW-vBPuMgTQ3c_0nlqHjW-WGqPwnYtlNjR8ObcwZMZIluRTyiUrw5xBNMeupVFzBSihmLaC7wZpYjaIEESW6GPUkgas9DOanv7EQxIGYnXjODMhxsZx_6SrNvQp1JEIc0GtGVHkvEXvXcGg==&uniplatform=NZKPT&language=CHS

[81] Ndreu L Hurben AK Nyman GSA Tretyakova NY Karlsson I Hagvall L. Investigation into propolis components responsible for inducing skin allergy: air oxidation of caffeic acid and its esters contribute to hapten formation. Chem Res Toxicol. (2023) 36:859–69. doi: 10.1021/acs.chemrestox.2c00386, PMID: 37184291 PMC10283018

[82] Chen JW Shen XG Hu FL. Bioconversion technology for reducing allergens in propolis. China Beekeeping. (2016) 67:62. Available online at: https://kns.cnki.net/kcms2/article/abstract?v=dTSX2bdXfeBPqFm9FHD9yXT8ZV0pJblwdh6AM9O130hlYvzuMKK92nqjP56meO4ea5YEUm7F4nGpfDCIHvannP9gjcQZROedYPrYDqo8ci_b1eK8Wm8X8ZRJqa1dJY1H238GrEm9OlbXL9wTNL4ZBWjvIs5Fe83jIsYkXYavfOp3m48TE6vhnQ==&uniplatform=NZKPT&language=CHS

[83] Rolver MG Emanuelsson F Nordestgaard BG Benn M. Contributions of elevated CRP, hyperglycaemia, and type 2 diabetes to cardiovascular risk in the general population: observational and Mendelian randomization studies. Cardiovasc Diabetol. (2024) 23:165. doi: 10.1186/s12933-024-02207-0, PMID: 38730445 PMC11088022

[84] Socha R Gałkowska D Bugaj M Juszczak L. Phenolic composition and antioxidant activity of propolis from various regions of Poland. Nat Prod Res. (2015) 29:416–22. doi: 10.1080/14786419.2014.949705, PMID: 25185953

[85] Cumpston M Li T Page MJ Chandler J Welch VA Higgins JP . Updated guidance for trusted systematic reviews: a new edition of the Cochrane handbook for systematic reviews of interventions. Cochrane Database Syst Rev. (2019) 10:ED000142. doi: 10.1002/14651858.ED000142, PMID: 31643080 PMC10284251

