# Supplementary material for: Propolis effects on blood sugar and lipid metabolism, inflammatory indicators, and oxidative stress in people with type 2 diabetes: a systematic review and meta-analysis
Source: Front Nutr. 2025 Oct 9;12:1653730. doi: 10.3389/fnut.2025.1653730 (PMC12545095; doi:10.3389/fnut.2025.1653730)
Supplement: Supplementary file 1 [file Table_1.docx]

The search terms include: propolis, Bee Propolis, Bee Glue, Bee Bread, diabetes, Diabetes Mellitus, type 2 diabetes, lipid metabolism, blood lipid, cholesterol, blood glucose, glucose metabolism, C-reactive protein, tumor necrosis factor-α, Interleukin-6, Malondialdehyde, superoxide dismutase, oxidative stress, oxidative damage, oxidative injury, antioxidative stress, anti-oxidative stress.

**Specific search strategy：**

Nine databases, including Chinese National Knowledge Infrastructure(CNKI), VIP Database, China Biomedical Literature Database(Sinomed), Wanfang Database, Pubmed, Cochrane Library, Embase, Scopus and Web of Science, were searched using the combination of MeSH terms and free words. The retrieval period was from the inception of the database to May 20, 2025.

1. **In Chinese databases, the search was conducted considering the CNKI database as an example of a retrieval strategy:**

**(SU='蜂胶' + '蜂胶黄酮') AND (SU='糖尿病' + '2型糖尿病') AND (SU='血脂' + '胆固醇' + '脂代谢' + '血糖' + '糖代谢' + '胰岛素抵抗' + '氧化应激' + '氧化损伤' + '肿瘤坏死因子-α' + 'c -反应蛋白' + '丙二醛' + '超氧化物歧化酶' + '白细胞介素-6')**

1. **In Chinese databases, the search was conducted considering the Wanfang database as an example of a retrieval strategy:**

**主题:(蜂胶 or 蜂胶黄酮) and 题名或关键词:(糖尿病 or 2型糖尿病) and 题名或关键词:(血脂 or 胆固醇 or 脂代谢 or 血糖 or 糖代谢 or 空腹血糖 or 胰岛素抵抗 or 肿瘤坏死因子-α or c -反应蛋白 or 丙二醛 or 超氧化物歧化酶 or 白细胞介素-6 or 氧化应激 or 氧化损伤)**

1. **In Chinese databases, the search was conducted considering the VIP database as an example of a retrieval strategy:**

**M=（蜂胶 OR 蜂胶黄酮) AND M=(血脂 OR 胆固醇 OR 脂代谢 OR 血糖 OR 糖代谢 OR** **胰岛素抵抗 OR 肿瘤坏死因子-α OR c -反应蛋白 OR丙二醛 OR 超氧化物歧化酶 OR** **白细胞介素-6 OR 氧化应激 OR 氧化损伤) AND M=(糖尿病 or 2型糖尿病)**

1. **In Chinese databases, the search was conducted considering the Sinomed database as an example of a retrieval strategy:**

**("蜂胶"[常用字段:智能] OR "蜂胶黄酮"[常用字段:智能]) AND ("糖尿病"[常用字段:智能] OR "2型糖尿病"[常用字段:智能]) AND ("血脂"[常用字段:智能] OR "胆固醇"[常用字段:智能] OR "脂代谢"[常用字段:智能] OR "血糖"[常用字段:智能] OR "糖代谢"[常用字段:智能] OR "氧化应激"[常用字段:智能] OR "c -反应蛋白"[常用字段:智能] OR "丙二醛"[常用字段:智能] OR "超氧化物歧化酶"[常用字段:智能] OR "白细胞介素-6"[常用字段:智能] OR "肿瘤坏死因子-α"[常用字段:智能****] OR "氧化损伤"[常用字段:智能])**

1. **For English databases, Pubmed was used as an example:**#1 AND #2 AND #3

#1**(((Propolis[MeSH Terms]) OR (Propolis[Title/Abstract])) OR (bee bread[Title/Abstract])) OR (bee glue[Title/Abstract])**

#2 **((diabetes mellitus[MeSH Terms]) OR (type 2 diabetes[Title/Abstract])) OR (diabetes[Title/Abstract])**

#3 **(((((((((((((lipid metabolism[Title/Abstract]) OR (blood lipid[Title/Abstract])) OR (cholesterol[Title/Abstract])) OR (blood glucose[Title/Abstract])) OR (glucose metabolism[Title/Abstract])) OR (Interleukin-6[Title/Abstract])) OR (Malondialdehyde[Title/Abstract])) OR (c-reactive protein[Title/Abstract])) OR (tumor necrosis factor-α[Title/Abstract])) OR (superoxide dismutase[Title/Abstract])) OR (oxidative stress[Title/Abstract])) OR (oxidative damage[Title/Abstract])) OR (oxidative injury[Title/Abstract])) OR (Antioxidative Stress[Title/Abstract])**

1. **For English databases, Embase was used as an example:** #1 AND #2 AND #3

#1 ：

**'propolis'/exp OR 'propolis' OR 'bee propolis':ti,ab,kw OR 'bee bread':ti,ab,kw OR 'bee glue':ti,ab,kw**

#2：

**'lipid metabolism'/exp OR 'lipid metabolism' OR 'blood lipid':ti,ab,kw OR 'cholesterol':ti,ab,kw OR 'blood glucose':ti,ab,kw OR 'glucose metabolism':ti,ab,kw OR 'interleukin-6':ti,ab,kw OR 'malondialdehyde':ti,ab,kw OR 'c-reactive protein':ti,ab,kw OR 'tumor necrosis factor-α':ti,ab,kw OR 'superoxide dismutase':ti,ab,kw OR 'oxidative stress':ti,ab,kw OR 'oxidative damage':ti,ab,kw OR 'oxidative injury':ti,ab,kw OR 'antioxidative stress':ti,ab,kw OR 'anti-oxidative stress':ti,ab,kw**

#3：

**'diabetes mellitus'/exp OR 'diabetes mellitus' OR 'diabetes mellitus':ti,ab,kw OR 'type 2 diabetes':ti,ab,kw OR 'diabetes':ti,ab,kw**

1. **For English databases, Web of science was used as an example:** #1 AND #2

#1：

**TS=(** **propolis OR** **Bee Propolis OR** **Bee Bread OR Bee Glue)**

#2：

**TS=( diabetes mellitus OR type 2 diabetes OR diabetes)**

#3：

**TS=(****lipid metabolism OR** **Blood lipid OR** **cholesterol OR blood glucose** **OR glucose metabolism OR** **oxidative stress OR oxidative damage OR oxidative injury OR Antioxidative Stress OR** **Interleukin-6 OR Malondialdehyde** **OR C-reactive protein OR tumor necrosis factor-α OR superoxide dismutase)**

1. **For English databases, The Cochrane library was used as an example:** #1 AND #2 AND #3

#1：

**((propolis):ti,ab,kw OR (Bee Propolis):ti,ab,kw OR (Bee Bread):ti,ab,kw OR (Bee Glue):ti,ab,kw)**

#2：

**((diabetes mellitus):ti,ab,kw OR (type 2 diabetes):ti,ab,kw OR (diabetes):ti,ab,kw)**

#3：

**((lipid metabolism):ti,ab,kw OR (Blood lipid):ti,ab,kw OR (cholesterol):ti,ab,kw OR (blood glucose):ti,ab,kw OR (glucose metabolism):ti,ab,kw OR (oxidative stress):ti,ab,kw OR (oxidative damage):ti,ab,kw OR (oxidative injury):ti,ab,kw OR (Antioxidative Stress):ti,ab,kw OR (Anti-oxidative Stress):ti,ab,kw) OR (Interleukin-6):ti,ab,kw OR (Malondialdehyde):ti,ab,kw OR (C-reactive protein):ti,ab,kw OR (tumor necrosis factor-α):ti,ab,kw OR (superoxide dismutase):ti,ab,kw）**

1. **For English databases, The Scoups was used as an example:** #1 AND #2 AND #3

#1：

**( TITLE-ABS-KEY ( "propolis" ) OR TITLE-ABS-KEY ( "Bee Propolis" ) OR TITLE-ABS-KEY ( "Bee Bread" ) OR TITLE-ABS-KEY ( "Bee Glue" ) )**

#2：

**( TITLE-ABS-KEY ( "Diabetes Mellitus" ) OR TITLE-ABS-KEY ( "type 2 diabetes" ) OR TITLE-ABS-KEY ( "diabetes" ) )**

#3：

**( TITLE-ABS-KEY ( "lipid metabolism" ) OR TITLE-ABS-KEY ( "blood lipid" ) OR TITLE-ABS-KEY ( "cholesterol" ) OR TITLE-ABS-KEY ( "blood glucose" ) OR TITLE-ABS-KEY ( "glucose metabolism" ) OR TITLE-ABS-KEY ( "Interleukin-6" ) OR TITLE-ABS-KEY ( "Malondialdehyde" ) OR TITLE-ABS-KEY ( "C-reactive protein" ) OR TITLE-ABS-KEY ( "tumor necrosis factor-α" ) OR TITLE-ABS-KEY ( "superoxide dismutase" ) OR TITLE-ABS-KEY ( "oxidative stress" ) OR TITLE-ABS-KEY ( "oxidative damage" ) OR TITLE-ABS-KEY ( "oxidative injury" ) OR TITLE-ABS-KEY ( "antioxidative stress" ) OR TITLE-ABS-KEY ( "anti-oxidative stress" ) )**
